# The HD-GYP Domain Protein RpfG of *Xanthomonas oryzae* pv. *oryzicola* Regulates Synthesis of Extracellular Polysaccharides that Contribute to Biofilm Formation and Virulence on Rice

**DOI:** 10.1371/journal.pone.0059428

**Published:** 2013-03-27

**Authors:** Yuanbao Zhang, Chao Wei, Wendi Jiang, Lei Wang, Churui Li, Yunyue Wang, John Maxwell Dow, Wenxian Sun

**Affiliations:** 1 Department of Plant Pathology and the Ministry of Agriculture Key Laboratory for Plant Pathology, China Agricultural University, Beijing, China; 2 Ministry of Education Key Laboratory for Agriculture Biodiversity and Plant Disease Management, Yunnan Agricultural University, Kunming, China; 3 BIOMERIT Research Centre, Department of Microbiology, BioSciences Institute, University College Cork, Cork, Ireland; University of the West of England, United Kingdom

## Abstract

Bacterial leaf streak caused by *Xanthomonas oryzae* pv. *oryzicola* (*Xoc*) is one of the most important diseases in rice. However, little is known about the pathogenicity mechanisms of *Xoc*. Here we have investigated the function of three HD-GYP domain regulatory proteins in biofilm formation, the synthesis of virulence factors and virulence of *Xoc.* Deletion of *rpfG* resulted in altered production of extracellular polysaccharides (EPS), abolished virulence on rice and enhanced biofilm formation, but had little effect on the secretion of proteases and motility. In contrast, mutational analysis showed that the other two HD-GYP domain proteins had no effect on virulence factor synthesis and tested phenotypes. Mutation of *rpfG* led to up-regulation of the type III secretion system and altered expression of three putative glycosyltransferase genes *gumD, pgaC* and *xagB*, which are part of operons directing the synthesis of different extracellular polysaccharides. The *pgaABCD* and *xagABCD* operons were greatly up-regulated in the *Xoc ΔrpfG* mutant, whereas the expression of the *gum* genes was unaltered or slightly enhanced. The elevated biofilm formation of the *Xoc ΔrpfG* mutant was dramatically reduced upon deletion of *gumD*, *xagA* and *xagB,* but not when *pgaA* and *pgaC* were deleted. Interestingly, only the *ΔgumD* mutant, among these single gene mutants, exhibits multiple phenotype alterations including reduced biofilm and EPS production and attenuated virulence on rice. These data indicate that RpfG is a global regulator that controls biofilm formation, EPS production and bacterial virulence in *Xoc* and that both *gumD-* and *xagB*-dependent EPS contribute to biofilm formation under different conditions.

## Introduction

*Xanthomonas oryzae* pv. *oryzicola* (*Xoc*) causes bacterial leaf streak (BLS) in rice, one of the most important bacterial diseases in tropical and subtropical Asia, some parts of Africa, as well as rice-growing regions of northern Australia. The BLS disease can cause yield loss up to 30% in epidemic years [1], [2], [3]. *Xoc* invades rice leaves mainly through stomata, and sometimes through wounds. The pathogen colonizes in the intercellular spaces of the parenchyma and is restricted to the apoplast of the mesophyll tissue. *Xoc* does not invade the xylem, which is in contrast to another rice bacterial pathogen *Xanthomonas oryzae* pv. *oryzae* (*Xoo*) that causes bacterial blight by invading vascular tissues [2]. Interactions between *Xoo*/*Xoc* and rice have become models for understanding fundamental aspects of bacterial pathogenesis in host plants and plant disease resistance, as well as functional and comparative genomics in microbial biology [4].

Several categories of genes have been identified to contribute to *Xoc* virulence; these include genes encoding functions involved in type III secretion, lipopolysaccharide synthesis, type IV pilus and twitching motility, carbohydrate synthesis and two-component regulation [5], [6]. Further to this, comparative genomic studies have revealed the conservation of functions or genes with an established role in virulence in other *Xanthomonas* species within the *Xoc* genome. Of particular interest here are proteins implicated in intracellular signaling involving the nucleotide second messenger cyclic diguanosine monophosphate (c-di-GMP), which has been implicated in virulence of a number of xanthomonads as well as a diverse range of unrelated bacterial pathogens [7], [8], [9], [10].

c-di-GMP was first identified in *Gluconacetobacter xylinus* as an activator of cellulose synthesis [11]. The molecule was later identified to be a widely conserved second messenger that is implicated in the regulation of various biological functions in bacteria, such as cellulose biosynthesis [11], bacterial motility [12], biofilm formation [13], the production of extracellular polysaccharide and the secretion of extracellular hydrolytic enzymes such as proteases and endoglucanases [14], [15], [16], [17]. The level of c-di-GMP in bacteria is regulated by at least three categories of proteins containing GGDEF, EAL or HD-GYP domains, respectively [8], [18], [19]. GGDEF domain-containing proteins function as diguanylate cyclases (DGCs) that synthesize c-di-GMP [18], while the HD-GYP and EAL domain-containing proteins act as phosphodiesterases (PDEs) that degrade c-di-GMP [7], [8], [19], [20]. All three domains are broadly distributed in many bacterial species [21].

A total of 37 proteins with HD-GYP, GGDEF and/or EAL domains were identified in the genome of *Xcc* 8004 [22]; *Xoo* PXO99 and *Xoc* BLS256 genomes encode at least 27 and 32 such proteins, respectively [23], [24]. The contribution of all 37 proteins to *Xcc* virulence has been examined by a functional genomic approach [22]. The findings showed that many proteins with GGDEF and/or EAL domains in addition to the HD-GYP domain protein RpfG contributed to *Xcc* virulence in Chinese radish. In *Xoo*, two groups have reported the involvement of proteins carrying GGDEF and EAL domains in motility, biofilm formation and/or virulence [10], [25]. To our knowledge, however, no functional studies for GGDEF, EAL or HD-GYP domain proteins have been reported in *Xoc* so far. A greater understanding of c-di-GMP signaling system in *Xoc* and its role in the interactions between the pathogen and host rice plants could have substantial implications for new approaches for disease control.

All *Xanthomonas* spp. genomes encode three conserved HD-GYP domain proteins of which the best studied is RpfG of *Xcc*. The RpfG regulator comprises a CheY-like receiver domain and an HD-GYP domain and acts together with the sensor kinase RpfC in a two-component system implicated in sensing and transduction of the diffusible signal factor DSF [26], [27]. The synthesis of DSF is dependent on RpfF, which belongs to the crotonase family and is encoded by a linked gene [17], [22], [26]. RpfG, which is required for full virulence of *Xcc,* positively regulates motility and the synthesis of virulence determinants such as extracellular polysaccharide (EPS) and extracellular enzymes but negatively regulates biofilm formation [17], [22], [26], [27], [28]. The regulatory action of this protein on extracellular enzyme synthesis depends upon the c-di-GMP phosphodiesterase of the HD-GYP domain whereas the influence on motility depends upon the interaction of RpfG with two GGDEF domain proteins, directed by the GYP motif of the HD-GYP domain [8], [29], [30]. The two other HD-GYP domain proteins in *Xcc* 8004 are XC0362 and XC1755. Deletion of *XC1755* attenuated virulence of *Xcc* on Chinese radish but had no effect on the secretion of extracellular enzymes whereas mutation of *XC0362* had no effect on virulence or extracellular enzyme production [22], indicating that HD-GYP domain proteins have diverse actions. The *Xoc* BLS256 genome encodes three HD-GYP domain proteins; XOC2264 (RpfG), XOC1984, and XOC4564 share 95.2%, 86.0% and 82.6% sequence identity to *Xcc* HD-GYP proteins XC2335 (RpfG), XC1755 and XC0362, respectively. Although RpfG is well studied in *Xcc*, little is known for the function of HD-GYP domain proteins in other phytopathogenic bacteria.

Here we describe experiments to address the function and regulatory role of HD-GYP domain proteins in *Xoc* by examination of the effects of deletion of the encoding genes. We directly tested virulence to rice as well as effects on production of a range of virulence factors including biofilm formation, extracellular enzyme production and expression of type III secretion systems (T3SS). We show that RpfG is essential for full virulence in *Xoc* and has a substantial influence on biofilm formation and EPS production, although only a minor effect on the secretion of extracellular proteases and swimming motility. Subsequent expression and functional analyses demonstrated that expression of three putative glycosyltransferase genes *gumD*, *xagB* and *pgaC* was differentially regulated by RpfG and that GumD and XagB are important factors for biofilm formation in *Xoc.*

## Results

### The *Xoc* Genome Encodes three HD-GYP Domain Proteins

The complete genome sequence of *Xoc* strain BLS256 allowed us to identify the HD-GYP domain proteins in *Xoc* through bioinformatic analysis [23]. BLAST searches revealed that three genes encode HD-GYP domain proteins in *Xoc* BLS256; these are *XOC2264* (*rpfG* ), *XOC1984*, and *XOC4564*. The latter two proteins were designated as HgdA and HgdC (HD-GYP domain-containing proteins). RpfG and HgdA have a CheY-like response receiver (REC) regulatory domain at N-terminus, whereas in HgdC, the HD-GYP domain comprises the central region of the un-characterized protein (Figure S1). To determine the effect of these HD-GYP domain proteins on bacterial behaviors and *in vivo* virulence of *Xoc*, the single, double and triple mutants involving *rpfG*, *hgdA* and *hgdC* genes were constructed as described in Materials and Methods and confirmed by Southern blot analyses (Figure S2).

### Mutations of *hgdA*, *rpfG* and *hgdC* Genes have Distinct Effects on Biofilm Formation in *Xoc*

**Figure 1:**
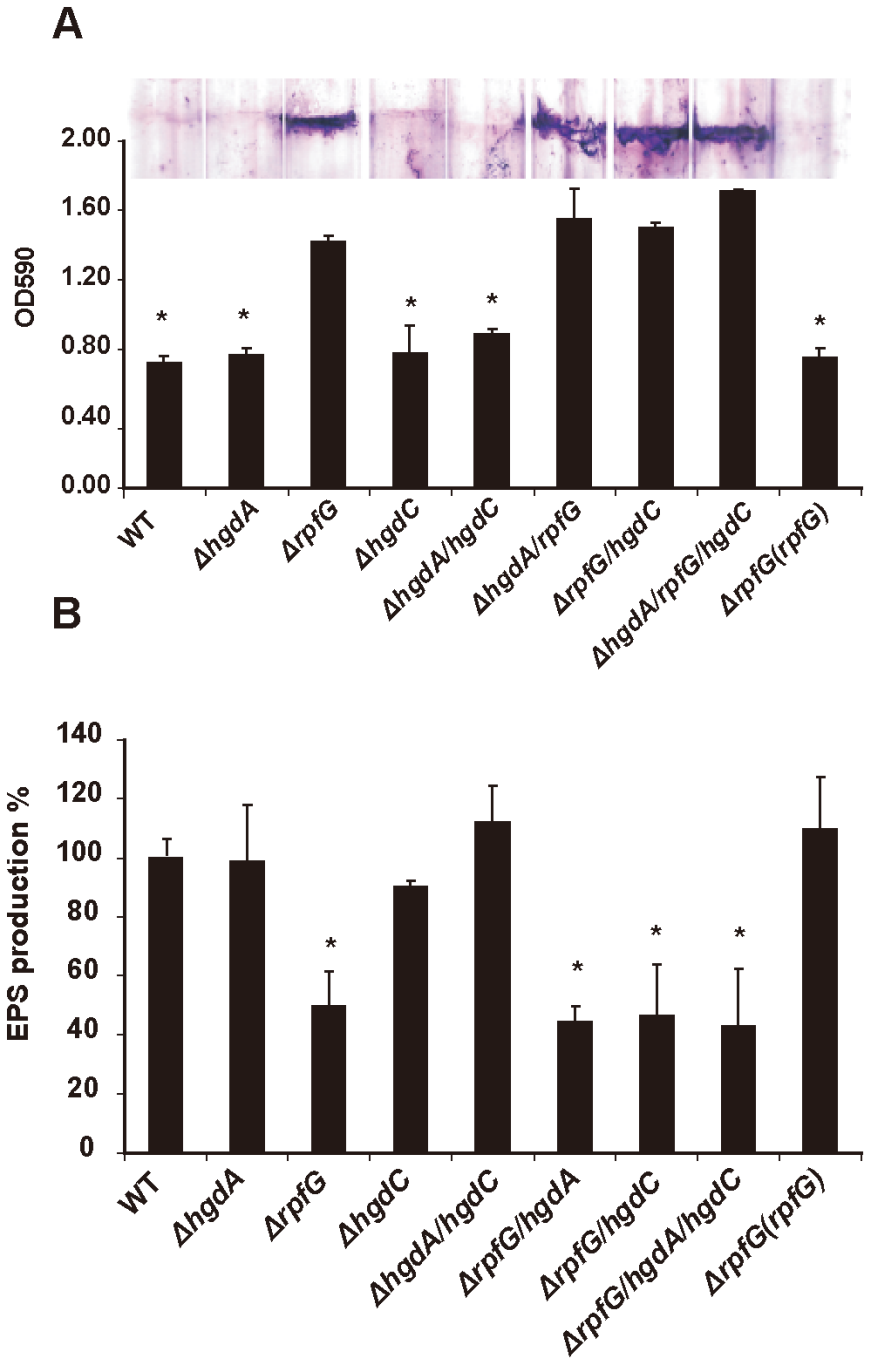
Effects of *hgdA*, *rpfG* and *hgdC* mutations on biofilm formation and the production of extracellular polysaccharide (EPS) in *X. oryzae* pv.*oryzicola*. (A) Increased biofilm formation was observed after mutation of *rpfG,* but not *hgdA* and *hgdC* deletion. Introducing the full-length *rpfG* gene into *ΔrpfG* reduced biofilm production to the wild-type level. Upper panel: The glass-bound biofilm was stained with crystal violet; Lower panel: The crystal violet in the stained biofilm on glass tubes was dissolved with 90% ethanol and detected spectrophotometrically at 590 nm. (B) EPS production was reduced when *rpfG,* but not *hgdA* and *hgdC,* was deleted in *Xoc*. The complemented strain *ΔrpfG(rpfG)* had a level of EPS production similar to the wild-type.

RpfG and c-di-GMP are important regulatory factors in biofilm formation in those xanthomonad bacteria tested thus far [22], [30], [31]. To evaluate the function of HD-GYP proteins in biofilm formation in *Xoc*, the wild-type and mutant strains were quantified for biofilm production at the air-media interface in glass tubes in L medium using crystal violet (CV) staining (see Methods). The *ΔrpfG* mutant produced approximately twice the amount of biofilm as the wild-type strain, whereas the *hgdA*, *hgdC* or the double *hgdA*/*hgdC* mutant strains produced wild-type levels of biofilm in L-medium (Figure 1A). The *rpfG* double and triple mutants involving *hgdA* and/or *hgdC* were not significantly different from the single *rpfG* mutant in biofilm production. Complementation analysis, which was performed by transforming the pVSP61 plasmid with the full-length *rpfG* gene into *ΔrpfG*, restored the phenotype of *ΔrpfG* in biofilm formation to the wild-type level (Figure 1A). The results suggest that under the conditions used, RpfG negatively regulates biofilm formation in *Xoc* but that HgdA and HgdC have no influence. Work in *Xcc* has shown that the diffusible signal molecule DSF also negatively regulates aggregation and biofilm formation through a pathway involving RpfG [27], [32]. Similarly, the *ΔrpfF* mutant of *Xoc*, which cannot synthesize DSF, produced much more biofilm when cultured in L-medium (Figure S3). The *Xoc rpfF* complementation strain restored the wild-type phenotype in biofilm formation (Figure S3).

### The Effects of *hgdA*, *rpfG* and *hgdC* Gene Deletions on the Production of Extracellular Polysaccharide, Protease Secretion and Motility in *Xoc*

Extracellular polysaccharide (EPS) is well known as one of important virulence factors in phytopathogenic bacteria [33]. To determine if RpfG and other HD-GYP domain proteins play a role in EPS production in *Xoc*, we quantified EPS produced in single, double and triple mutant strains with *hgdA*, *rpfG* and/or *hgdC* gene deletions. It was observed that the *ΔrpfG* mutant generated 53±9% of the EPS produced by the wild-type *Xoc*. However, the *ΔhgdA, ΔhgdC* and *ΔhgdA/hgdC* mutants produced similar amount of EPS to the wild-type strain (Figure 1B). The *ΔrpfG/hgdA, ΔrpfG/hgdC* and *ΔrpfG/hgdA/hgdC* mutant strains also produced much less EPS than the wild-type strain, but the levels in these strains were not significantly different from the *ΔrpfG* strain (Figure 1B). The ability to produce EPS of the *ΔrpfG* mutant was restored when a plasmid-borne full length *rpfG* gene was introduced into *ΔrpfG*.

Mutation of *rpfG* in *Xcc* caused a substantial reduction in the secretion of the extracellular enzymes endoglucanase, endomannanase and proteases [22]. *Xoc* secreted very low levels of endoglucanase and endomannanase after cell cultures were grown in NB and OB medium [34] (data not shown). By contrast, the secretion of proteases in *Xoc* can be easily detected by observing clearing zones around bacterial cultures in skim-milk-containing agar plates. As shown in Figure S4A, all single, double and triple deletion mutant strains exhibited no significant difference on the diameter of clearing zones, indicating that the ability to synthesize and secrete proteases is not altered in these mutants. Similar results were observed when these *Xoc* strains were cultured on the skim-milk NYGA plates (data not shown).

The swimming motility of the wild-type and different mutant strains was determined after inoculation of bacteria onto semi-solid plates. No mutant showed substantial alteration in its swimming motility from the wild-type (Figure S4B). These findings established that all of these HD-GYP domain proteins in *Xoc* are not involved in the synthesis and secretion of extracellular proteases and swimming motility. Taken together, the results indicate that RpfG positively regulates EPS production in *Xoc*, but has little or no influence on protease production or motility. The *hgdA* and *hgdC* genes have no effect on EPS production, motility or protease secretion when deleted either singly or in combination.

### Deletion of *rpfG*, but not of *hgdA* and *hgdC* Genes Reduces *Xoc* Virulence on Rice

**Figure 2:**
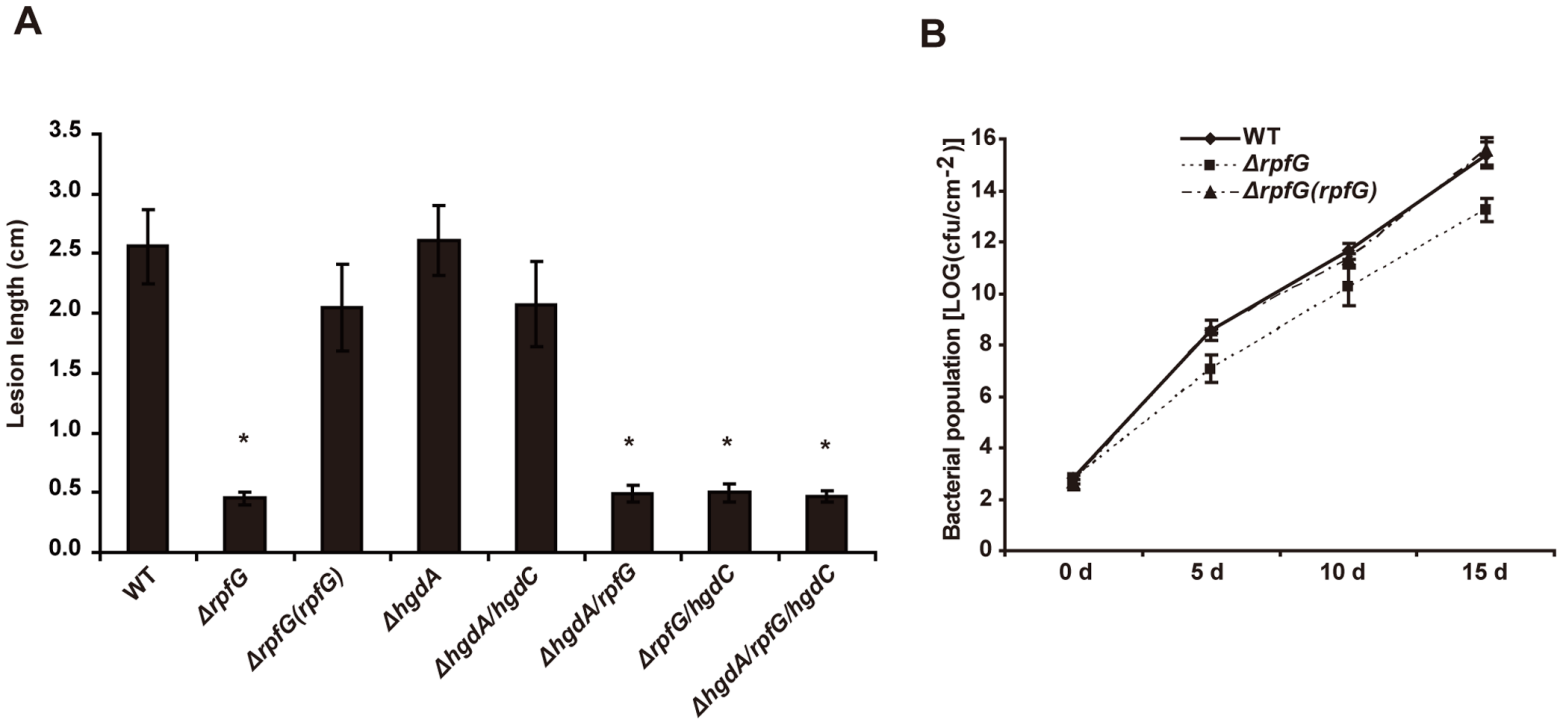
Virulence assays of the wild-type and *rpfG-, hgdA- and hgdC-*related single, double and triple mutant strains on rice cv. Jingang 30. (A) The length of disease lesions was measured at 14 days after pressure inoculation of the wild-type (WT), *ΔrpfG*, complemented *ΔrpfG (rpfG)*, *ΔhgdA*, *ΔhgdA/hgdC*, *ΔhgdA/rpfG*, *ΔrpfG/hgdC*, *ΔhgdA/rpfG/hgdC* strains, respectively. Ten to 15 leaves were scored for each strain; means ± standard error (SE) are shown. (B) *In planta* bacterial populations of *Xoc* RS105, *ΔrpfG* and *ΔrpfG(rpfG)* at the specific time points after inoculation. Data are presented as means ± SE.

To investigate the role of HD-GYP proteins in *Xoc* virulence on rice, the wild-type and mutant strains were pressure-inoculated into the leaves of six-week-old rice plants (*Oryza sativa* cvs. Nipponbare and Jingang 30). Virulence of each mutant was determined by measuring the length of disease lesions 2 weeks after inoculation. The deletion of *rpfG* caused nearly complete loss of *Xoc* virulence on rice, but the *hgdA* and *hgdC* gene deletions had no influence on *Xoc* virulence (Figure 2A). Complementation restored virulence of the *ΔrpfG* mutant towards the wild-type level. Enumeration of bacteria isolated from the inoculated rice leaves clearly showed that the *in planta* population size of *ΔrpfG* was much smaller than that of the wild-type and complemented strains (Figure 2B). The data indicate that RpfG plays an essential role in the virulence and colonization ability of *Xoc* on rice.

### The PDE Activity of RpfG is Required for Regulation of Virulence Factor Synthesis and Virulence to Rice

**Figure 3:**
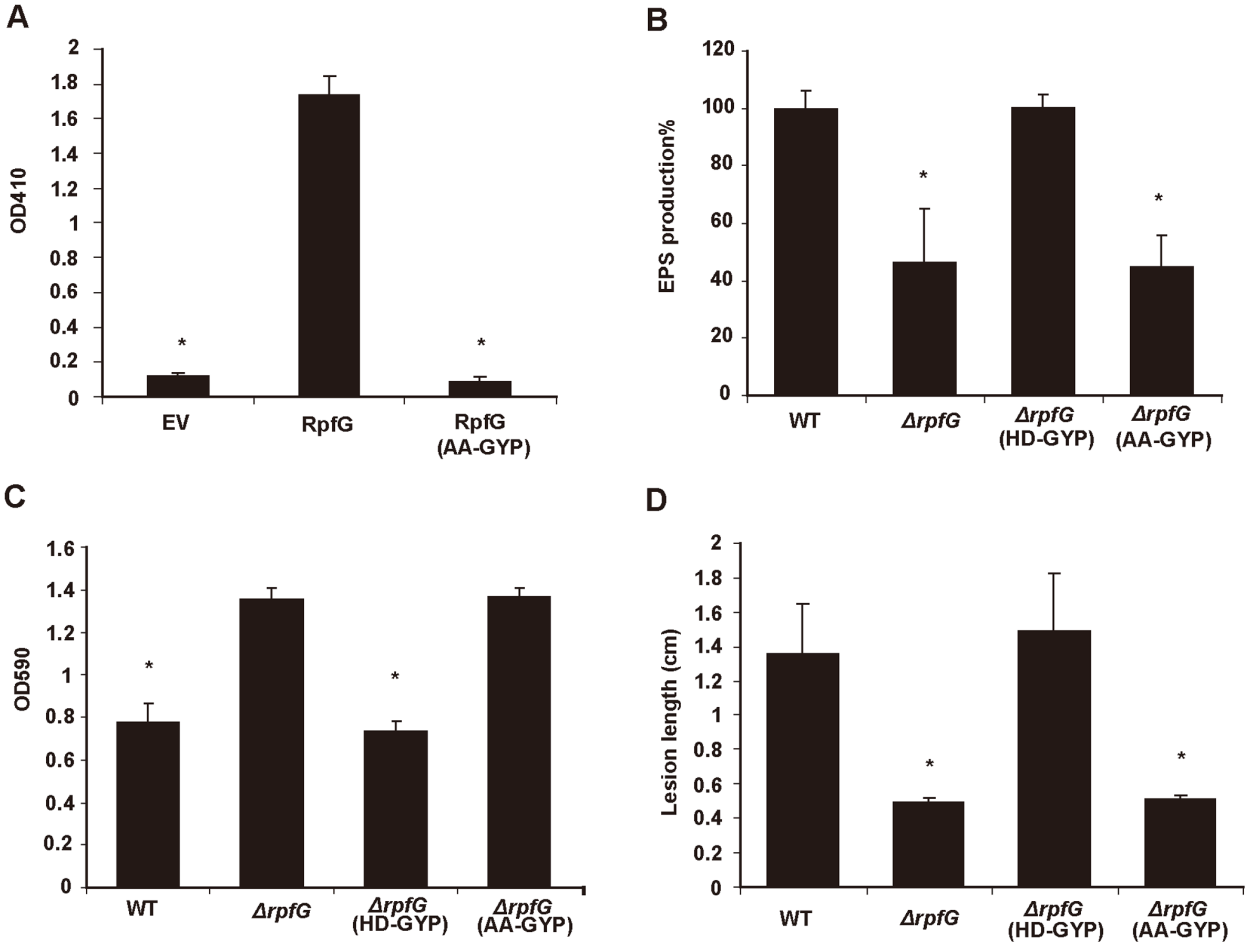
Mutation of H231 and D232 residues in the HD-GYP domain of *Xoc* RpfG disrupts the PDE activity and regulatory function. (A) Purified RpfG_AA-GYP_ completely lost its PDE activity detected by colorimetric assays. (B–D) RpfG_AA-GYP_ lost the ability to restore EPS production (B), biofilm formation (C) and virulence to rice (D) of *ΔrpfG* to the wild-type level. WT: wild-type, EV: empty vector.

The action of the HD-GYP domain as a PDE active against c-di-GMP has been demonstrated in proteins from several bacteria including *Xcc*, *Pseudomonas aeruginosa* and *Borrelia burgdorferi* [8], [9], [35]. To test whether the regulatory influence on virulence and virulence factor synthesis in *Xoc* depended upon the c-di-GMP PDE activity, our approach was to examine the effects of introducing alanine substitutions in the presumed HD catalytic diad [8], [36], [37], [38] on both the enzymatic and regulatory activities of the protein. RpfG was expressed in *E. coli* as N-terminal His6-tagged fusions and then purified using nickel columns (see Methods). The purified protein had the PDE activity against the model substrate bis(*p*- nitrophenyl) phosphate and could degrade c-di-GMP into two products identified by LC-MS as pGpG and GMP (Figure S5). The AA-GYP variant of RpfG, in which the residues of the HD diad were substituted by alanine, was also purified as an N-terminal His6-tag protein. As expected, this alteration of RpfG protein completely abolished its PDE activity as measured by the hydrolysis of *bis-*(p-nitrophenyl) phosphate (Figure 3A).

Then the importance of the active site mutation for RpfG regulatory function was assessed through comparative phenotype analyses of the *rpfG* mutant expressing either the wild-type (HD-GYP) protein or variant (AA-GYP) protein from the pVSP61 plasmid. The results showed that RpfG AA-GYP variant lost the ability to restore the mutant phenotypes of EPS production, biofilm formation and virulence on rice to the wild-type level (Figure 3B, 3C and 3D). The findings indicate that the regulatory influence of RpfG on virulence and virulence factor synthesis in *Xoc* depends upon the enzymatic activity against c-di-GMP, consistent with previous work on *Xcc* [8].

### RpfG Negatively Regulates T3SS Expression

**Figure 4:**
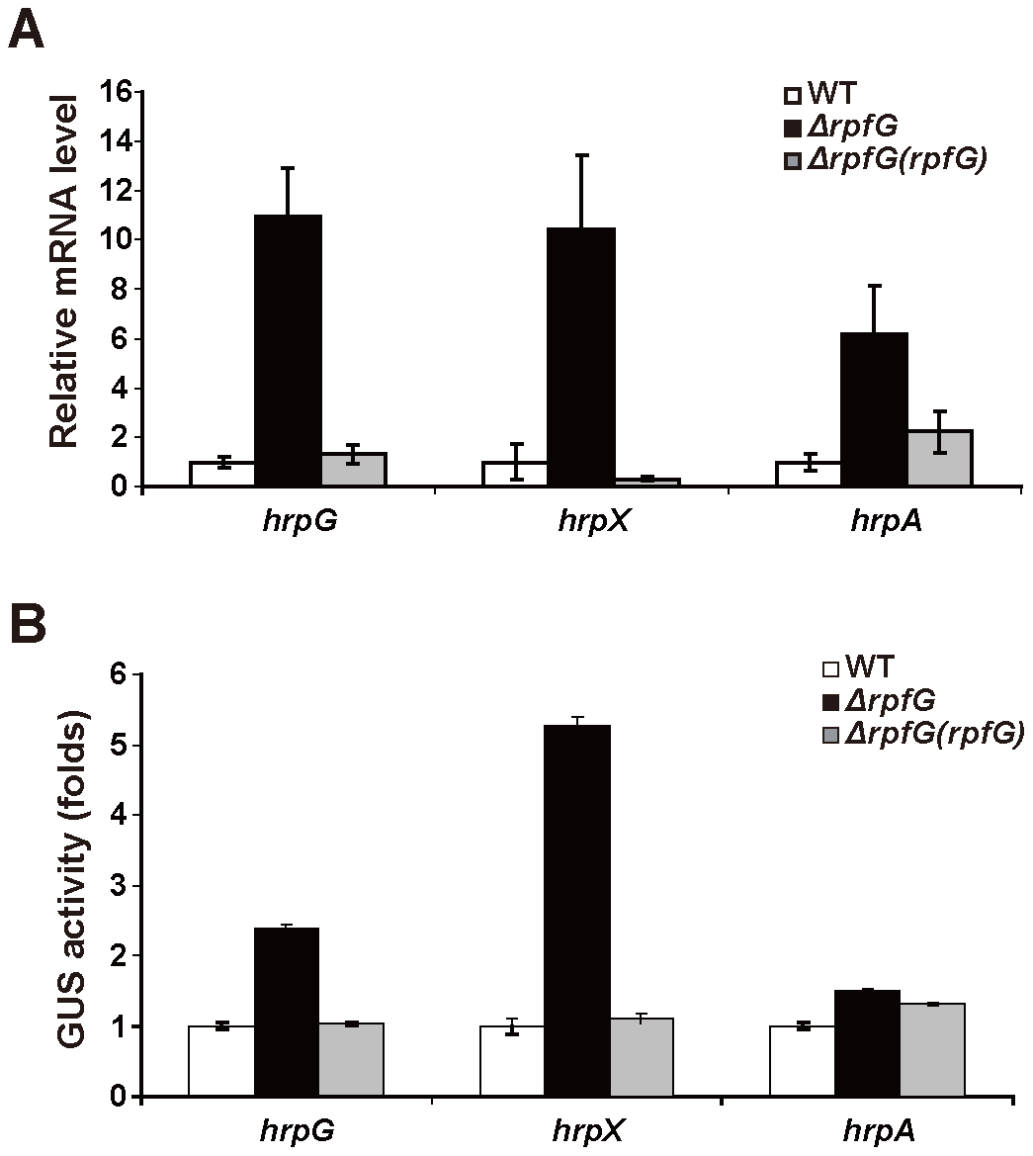
The effect of RpfG on the expression of *hrp*-related genes in *Xoc*. (A) Gene expression of *hrpG*, *hrpX* and *hrpA* in wild-type Xoc (RS105), *ΔrpfG* and complemented strain *ΔrpfG(rpfG)* was detected by qRT-PCR. 16S RNA was used as an internal control for data analyses. (B) Gene expression of *hrpG*, *hrpX* and *hrpA* in RS105, *ΔrpfG* and *ΔrpfG(rpfG)* strains was examined by GUS activities of appropriate promoter-GUS fusions. WT: wild-type.

The T3SS in most of plant pathogenic bacteria is up-regulated during host infection and essential for virulence [39], [40]. In *Dickeya dadantii*, expression of the T3SS genes *hrpA* and *hrpN* was dramatically reduced in *ΔecpB* and *ΔecpC*, suggesting a potential role of c-di-GMP in T3SS regulation [7]. To investigate if RpfG is also involved in the T3SS regulation in *Xoc*, the expression of three key *hrp* regulatory genes, *hrpG*, *hrpX* and *hrpA* in the wild-type and *ΔrpfG* mutant strains was examined using quantitative real-time polymerase chain reaction (qRT-PCR) (Figure 4A). A significant increase of *hrpG*, *hrpX* and *hrpA* mRNA expression was detected in *ΔrpfG*, compared to the wild-type and complementation strains. Furthermore, the expression of these genes in the *ΔrpfG* mutant was evaluated using the promotor-β-glucuronidase (GUS) fusions. It was demonstrated that the GUS activity driven by the *hrpX, hrpG* and *hrpA* promoter was up-regulated in *ΔrpfG* by about 2.5-fold, five-fold and 1.5-fold compared to the wild-type strain, respectively (Figure 4B). Complementation of *ΔrpfG* with full-length *rpfG* reduced expression of these genes towards the wild-type level (Figure 4). Thus both sets of expression data from qRT-PCR and *gusA* fusions indicate that RpfG negatively regulates the expression of these *hrp* regulatory genes. Notably, these gene expression analyses were investigated in XOM3 minimal medium [41], where the growth rate of *ΔrpfG* is similar to that of wild-type *Xoc* strain (Figure S6).

### RpfG has Divergent Effects on Expression of Genes Encoding Glycosyl Transferases

The *Xoc* genome encodes a number of putative glycosyltransferases, several of which have been demonstrated to be involved in biofilm formation in other bacteria. The *gum* operon, which is responsible for the synthesis of xanthan gum, has been shown to be involved in biofilm production in *Xcc* [42]. A modified gum cluster is required for biofilm formation in *Xylella fastidiosa* [43]. Deletion of a distinct gene cluster named *xag* in *Xcc* also resulted in decreased extracellular polysaccharide production and abolished biofilm formation [32]. In addition to *gum* and *xag* genes, the *Xoc* genome carries the *pgaABCD* operon, which is not found in *Xcc*. The *pga* operon directs the synthesis of poly-β-1,6-*N*-acetyl-D-glucosamine-like polysaccharide (β-1,6-GlcNAc; PGA), an extracellular polysaccharide that has been shown to serve as an adhesin and is required for biofilm formation in bacteria such as *Staphylococcus epidermidis* and *Escherichia coli* [44], [45], [46]. The effects of mutation of *rpfG* on biofilm formation, described above, prompted us to explore which of the three EPSs putatively produced by *Xoc* are important for biofilm formation.

**Figure 5:**
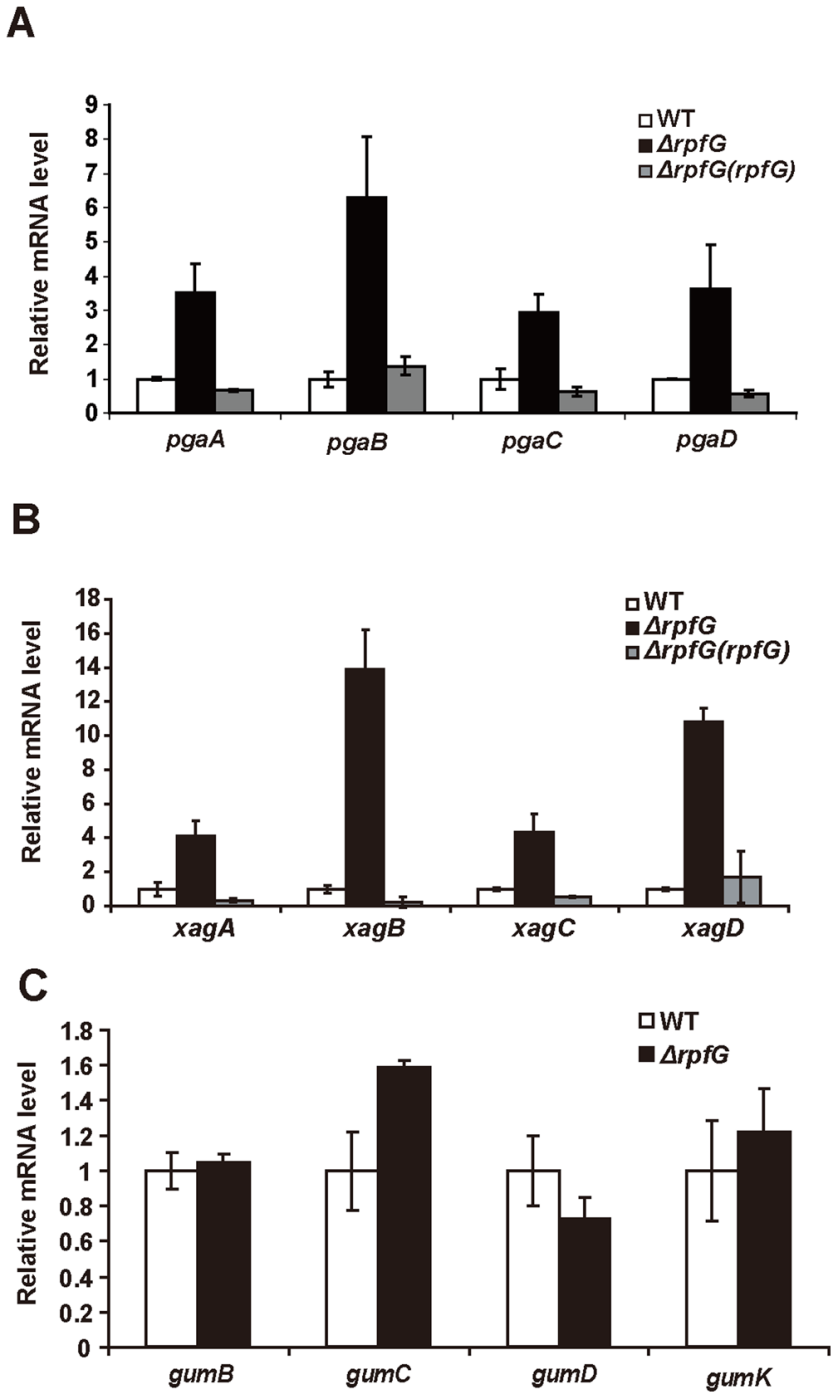
The effect of *rpfG* deletion on the expression of three putative glycosyltransferase gene operons, *pgaABCD*, *xagABCD* and the *gum* cluster in *Xoc*. (A–C) Gene expression of *pgaABCD* (A), *xagABCD* (B) and *gumB, gumC, gumD, gumK* (C) in wild-type *Xoc* RS105, *ΔrpfG* and complemented *ΔrpfG(rpfG)* strains was detected by qRT-PCR. 16S RNA was used as an internal control for data analyses.

As a first step towards this, the expression of *xag*, *pga* and *gum* operons in the wild-type and *rpfG* mutant backgrounds was quantified by qRT-PCR. The expression of *pga* and *xag* genes were up-regulated by 3 to 14 fold in *ΔrpfG* compared to the wild-type and complemented strains (Figure 5A and 5B). In contrast, the expression of all four tested *gum* genes was not dramatically altered or moderately up-regulated in the *Xoc ΔrpfG* mutant (Figure 5C). These data implied that RpfG has separate effects on expression of the *pgaABCD* and *xagABCD* operons and the *gum* cluster in *Xoc*.

### GumD and XagB, but not PgaC Contribute to Biofilm Formation in the *Xoc ΔrpfG* Mutant

**Figure 6:**
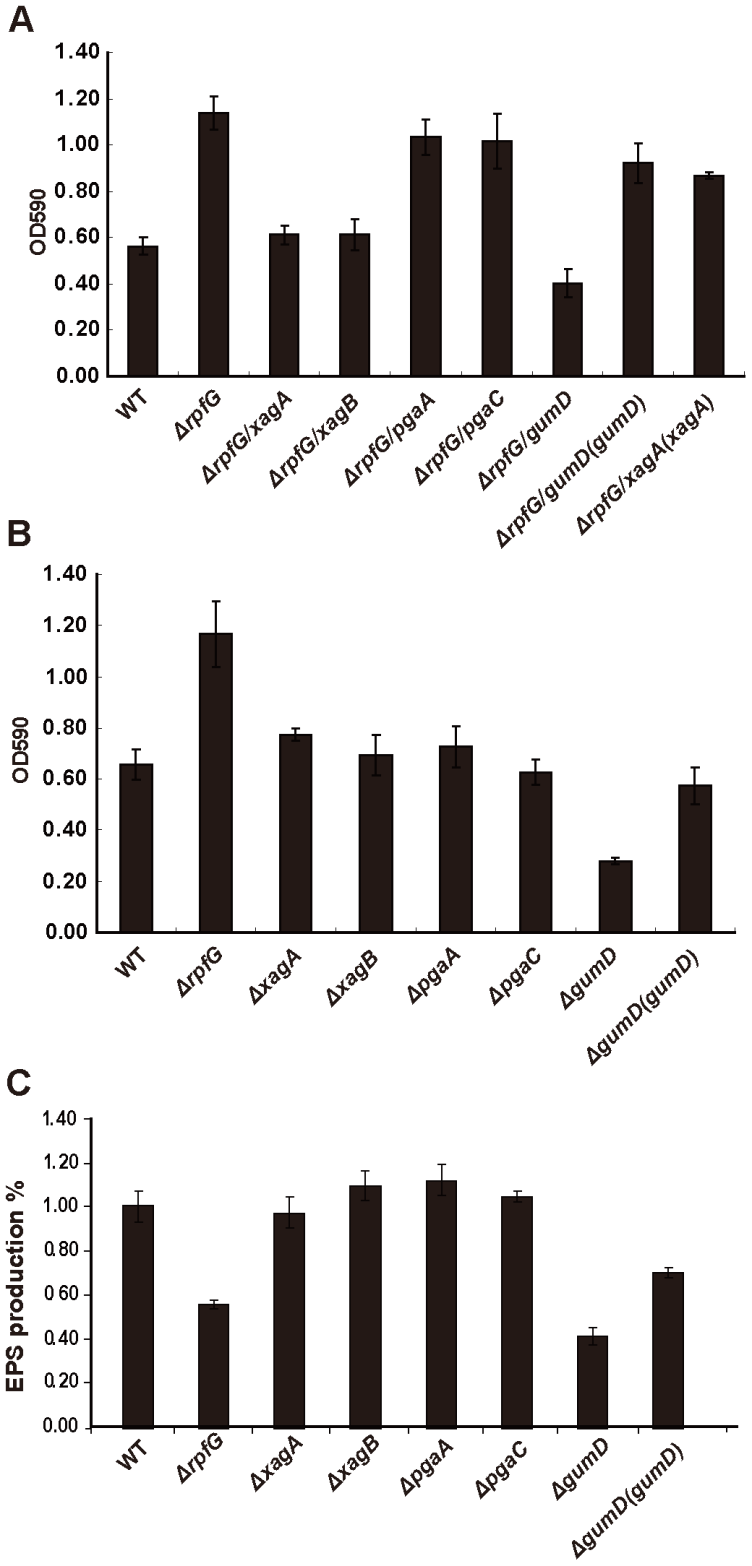
Effects of mutations of genes encoding several putative glycosyltransferases on biofilm formation and EPS production in *Xoc* wild-type and *ΔrpfG* mutant backgrounds. (A) Elevated biofilm formation in *Xoc ΔrpfG* was reduced to the wild-type level when *xagA*, *xagB* and *gumD*, but not *pgaA* or *pgaC* was deleted singly in the *ΔrpfG* genotype. The full length *xagA* and *gumD* genes restored the ability of biofilm formation in *ΔrpfG/xagA* and *ΔrpfG/gumD* mutants, respectively. (B) The ability of *ΔgumD* to form biofilm was greatly attenuated in the wild-type background, but was restored by complementation. Biofilm formation in the *ΔpgaA*, *ΔpgaC, ΔxagA* and *ΔxagB* single mutants was not altered compared to the wild-type strain. (C) EPS production was significantly reduced in the *ΔgumD* mutant, but not in *ΔpgaA*, *ΔpgaC, ΔxagA* and *ΔxagB* mutants compared to the wild-type. These experiments were repeated at least three times with similar results.

To determine which EPSs are essential for biofilm formation in *Xoc*, the genes *pgaA* and *pgaC* in the *pga* operon, *xagA* and *xagB* in the *xag* operon and *gumD* in the *gum* operon were deleted from the *Xoc* wild-type and *rpfG* mutant strains using homologous recombination. The ability to produce biofilm and EPS was investigated in all these mutant strains. As shown in Figure 6A, the *ΔrpfG/gumD, ΔrpfG/xagA* and *ΔrpfG/xagB* double mutants formed much less biofilm than the *ΔrpfG* single mutant, while the ability of *ΔrpfG/pgaA* and *ΔrpfG/pgaC* double mutants to produce biofilm is not altered compared to *ΔrpfG* mutant in L-medium. In a consistent fashion, the complemented strains in which the full-length *gumD* and *xagA* were introduced into the respective double mutants *ΔrpfG/gumD* and *ΔrpfG/xagA* restored the production of biofilm towards that seen in the single *rpfG* mutant (Figure 6A). These data indicate that the GumD- and XagB-dependent EPSs but not PGA contribute to elevated biofilm formation in the *rpfG* mutant.

To investigate the function of these three putative glycosyltransferases in biofilm formation in the wild-type background, we measured biofilm formed by the wild-type *Xoc* and single mutants in L-medium (Figure 6B). The single gene mutants *ΔpgaA*, *ΔpgaC*, *ΔxagA* and *ΔxagB* produced similar amount of biofilm to the wild-type strain, while the *ΔgumD* mutant exhibited much less adhesion to glass than the wild-type strain when cultured in L-medium (Figure 6B) and in NB medium (data not shown). Complementation to generate the *ΔgumD(gumD)* strain restored the phenotype near to the wild-type level. These data indicate that GumD-dependent xanthan plays an essential role in biofilm formation in both wild-type and *rpfG* mutant backgrounds.

Swimming motility of these single gene-deletion mutants was also tested and was not significantly altered compared to the wild-type (Figure S7). The contribution of the different polysaccharides to total EPS production was further investigated for *Xoc* strains grown in M210 medium that contains sucrose (see Methods). EPS produced in the *ΔpgaA*, *ΔpgaC*, *ΔxagA*, *ΔxagB* and *ΔgumD* single mutants was quantified and compared to the wild-type. The findings (Figure 6C) showed that only deletion of *gumD* greatly disrupted the ability of *Xoc* to produce EPS, although this ability can be partially restored by complementation (Figure 6C). EPS produced by the *ΔgumD* mutant is even less than that by the *ΔrpfG* mutant. This suggests that xanthan is by far the major EPS produced in *Xoc* under the conditions used for this experiment.

### GumD, XagB and PgaC have Differing Contributions to *Xoc* Virulence

**Figure 7:**
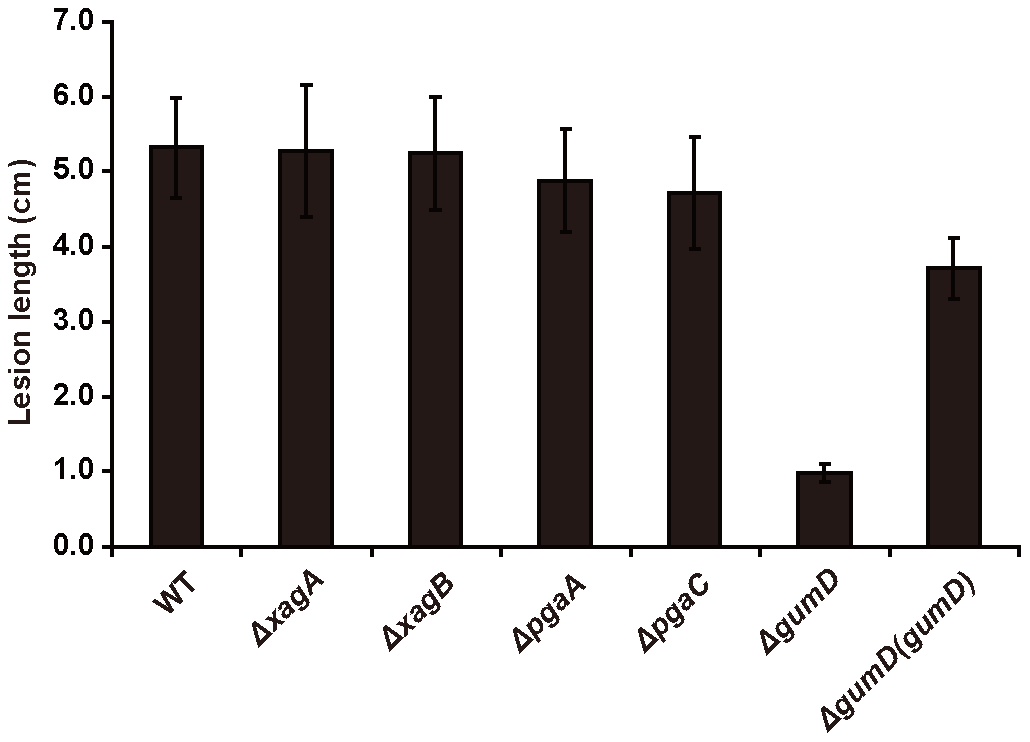
The effect of *gumD* deletion on bacterial virulence in *Xoc.* * Xoc* virulence to rice was greatly attenuated when *gumD* was deleted, but was unaltered in the *ΔpgaA*, *ΔpgaC, ΔxagA* and *ΔxagB* mutant strains. Virulence of the *ΔgumD* mutant was restored by the introduction of full-length *gumD* into the *ΔgumD(gumD)* strain. The length of disease lesions was measured at 20 days after pressure inoculation. Ten to 15 leaves were scored for each strain; means ± SE are shown. The experiments were repeated three times with similar results.

Besides the effect on EPS production and biofilm formation, we investigated the effect of deletions of the putative glycosyltransferase genes *gumD*, *xagB*, and *pgaC* on virulence to rice (Figure 7). Virulence of these deletion mutants was determined using pressure inoculation into rice leaves. Independent repeated experiments showed that virulence of the *gumD* mutant was greatly attenuated while virulence of other mutants including *xagA*, *xagB*, *pgaA* and *pgaC* was not altered (Figure 7).

## Discussion

Although more than 590 HD-GYP domain proteins in over 140 bacterial genomes have been reported, only a few have been characterized so far [8], [9], [47], [48]. RpfG in *Xcc*, which is one of the best-studied HD-GYP proteins, functions as a phosphodiesterase to break the signal molecule c-di-GMP and subsequently regulates various biological processes and bacterial virulence [8], [26], [27]. In the present study, we investigated the function of the three HD-GYP domain proteins (HgdA, RpfG and HgdC) in the important rice pathogen *Xoc*.

Deletion of *hgdA* and *hgdC* in *Xoc* had no effect on virulence to rice or on any of the phenotypes tested under our experimental conditions. In contrast, deletion of *rpfG* in *Xoc* resulted in decreased bacterial virulence to rice, increased biofilm formation and alterations in the expression of T3SS genes and genes encoding enzymes involved in the synthesis of different EPSs. The effects of *rpfG* mutation on virulence, synthesis of EPS and expression of *xag* and *gum* genes are similar to those seen in *Xcc*. However, the deletion of *rpfG* has no influence on swimming motility and the secretion of extracellular proteases in *Xoc*, while RpfG in *Xcc* positively regulates pilus-dependent motility and the secretion of extracellular enzymes including endoglucanase, endomannase and proteases [26], [27]. RpfG proteins in *Xoc* and *Xcc* are highly conserved (95.2% amino acid identical), both are active in c-di-GMP degradation and their regulatory activity depends upon their enzymatic activity. Thus differences in the regulatory functions of RpfG may reflect differences in the complement or action of c-di-GMP effectors in the two bacteria. Similar considerations may explain recent observations that DSF signaling system might regulate virulence-associated traits in a completely contrasting pattern in *Xoo* and *Xcc* [22], [27], [49].

Mutation of *rpfG* led to different effects on expression of three operons directing the synthesis of different EPSs. The expression of *xag* and *pga* genes was significantly up-regulated in *ΔrpfG* mutant, whereas the expression of *gum* genes was slightly increased or unaltered in the mutant (Figure 5). Consistent with our observations, comparisons of RpfF, RpfC and RpfG regulons in *X. citri* subsp. *citri* revealed that *XAC3522-XAC3525* genes that share high sequence identities with *xagABCD* in *Xoc* were up-regulated in *ΔrpfG* mutant [40]. Deletions of *rpfF*, *rpfC* or *rpfG* genes in *Xcc* led to drastically increased expression of *xagABCD* [32]. It is interesting to note that distribution of these different operons in different *Xanthomonas* spp. Comparative genomics reveals that although the *gum*, *xagABCD* and *pgaABCD* operons are all found in the *Xoc* genome, *xagABCD* is not found in *Xoo* and *pgaABCD* is not found in *Xcc*. Wider distribution of the *gum* operon implies that the *gum*-dependent EPS is likely more important to biofilm formation and other virulence-associated traits. Extensive phenotype analyses of multiple single and double mutants confirmed this hypothesis; *gumD* is critical for biofilm formation, EPS production and virulence under the wild-type and *ΔrpfG* mutant background (Figure 6 and 7). Further functional analyses showed that the deletion of *xagA* and *xagB* had different effects on biofilm formation under the *ΔrpfG* mutant and wild-type background (Figure 6A and 6B). The *ΔrpfG/pgaA* and *ΔrpfG/pgaC* double mutants exhibited the phenotype of *ΔrpfG* mutant in biofilm formation. The results indicate that both *gum-* and *xag-*dependent EPSs contribute to elevated adhesion in the *Xoc ΔrpfG* mutant. In *Pseudomonas aeruginosa,* at least three exopolysaccharides (alginate, Psl, and Pel) contribute to the formation of biofilms [50]. In *E. coli* cells, the glycosyltransferase PgaC is required for the synthesis of PGA that functions as an adhesin for biofilm formation [46]. Gene deletion analyses demonstrated that PgaC plays a minor role in biofilm formation in *Xoc*, suggesting that other adhesins present in this pathogen might have a role.

The regulatory activity of RpfG on biofilm formation and virulence factor synthesis in both *Xoc* and *Xcc* depends upon its action against c-di-GMP (Figure 3) and mutation of *rpfG* leads to an increase in the level of the nucleotide in *Xcc* [8]. The enzymatic action of RpfG is modulated by phosphorylation during sensing and transduction of the DSF signal. We speculate that similar changes in c-di-GMP level that are seen in the *rpfG* mutant can occur in the wild-type as a response to the presence (or absence) of different environmental cues that include DSF.

Deletion of *rpfG* in *Xoc* resulted in nearly complete loss of bacterial virulence despite unaltered bacterial motility and protease secretion and enhanced biofilm formation. A previous study in *D. dadantii* 3937 showed that both c-di-GMP PDEs, EcpB and EcpC, were required for the expression of the type III secretion system (T3SS) that is essential for bacterial virulence [7]. Reduced expression of T3SS is hypothesized to be one of the factors responsible for reduced bacterial virulence in the *ecpB* and *ecpC* mutants of *D. dadantii* 3937. Therefore, we investigated the expression of T3SS regulatory genes in *Xoc* wild-type and *ΔrpfG* mutant strains. HrpG, an OmpR family response regulator, controls the expression of *hrpA* to *hrpF* and *hrpX* in *X. campestris* pv. *vesicatoria* [51], [52] or only *hrpX* in *Xcc* [53]. HrpX, an AraC-type regulator, activates the expression of the *hrpB* operon and several effector genes [52], [54], [55]. Both GUS-fusion transcriptional assays and qRT-PCR results showed *hrpG*, *hrpX* and *hrpA* genes were all up-regulated in the *Xoc rpfG* mutant when cultured in minimal media XOM3. Paradoxically, although the *Xoc rpfG* mutant had elevated expression of T3SS, its virulence to rice was attenuated. We speculate that RpfG has such a broad regulatory influence on the cell that up-regulation of some factors such as T3SS cannot compensate for the down-regulation of others or override the negative effect of sustained biofilm formation. In *Xcc*, the expression of some *hrp* genes was also down-regulated by RpfF and diffusible siganl factor (DSF) under *in vitro* culture conditions [39]. In contrast, transcriptome analysis of RpfG regulon indicated that a few genes encoding the T3SS translocon and effectors were up-regulated by RpfG in *X. citri* subsp. *citri* [40]. However, these experiments were performed in rich medium where *hrp* genes are not fully expressed therefore it is comparison with the work described here on *Xoc* grown in XOM3 minimal medium should be made cautiously.

Identification of some aspects of the function of RpfG in *Xoc*, to include effects on EPS production and biofilm formation, increases our understanding of the c-di-GMP signaling in the regulation of virulence and virulence-associated traits. Nevertheless our picture of the roles of RpfG in regulation of bacterial virulence in *Xoc* is still far from complete.

## Materials and Methods

### Plant Materials, Bacterial Strains, Plasmids and Culture Conditions

Rice plants (*Oryza sativa* cvs. Nipponbare and Jingang 30) were grown in greenhouse. Bacterial strains and plasmids used in this study are listed in Table 1. The *Xoc* RS105 wild-type and mutant strains were grown in NB medium (beef extract, 3 g/L; yeast extract, 1 g/L; tryptone, 5 g/L; sucrose, 10 g/L ), NYG medium or in XOM3, T3SS-inducing minimal medium [56] at 28°C. Antibiotics were used at the following concentrations: ampicillin, 100 µg/ml; kanamycin, 50 µg/ml; rifampin (Rif), 25 µg/ml. All the experiments were repeated at least three times with similar results unless noted.

**Table 1:** Bacterial strains and plasmids.

Strains/plasmids	Characteristics	References or source
***E. coli***		
DH5α	High efficiency transformation	
pRK600	Helper strain in tri-parental mating	
***X. oryzae***		
RS105	Wild-type, Rif^R^	
*ΔhgdA*	In frame deletion of *XOC_1984*, Rif^R^	This Study
*ΔrpfG*	In frame deletion of *rpfG*, Rif^R^	This Study
*ΔhgdC*	In frame deletion of *XOC_4564*, Rif^R^	This Study
*ΔhgdA/rpfG*	In frame deletion of *XOC_1984* and *rpfG*, Rif^R^	This Study
*ΔhgdA/hgdC*	In frame deletion of *XOC_1984* and *XOC_4564*, Rif^R^	This Study
*ΔrpfG/hgdC*	In frame deletion of *rpfG* and *XOC_4564*, Rif^R^	This Study
*ΔhgdA/rpfG/hgdC*	In frame deletion of *XOC_1984*, *rpfG* and *XOC_4564*, Rif^R^	This Study
*ΔgumD*	In frame deletion of *gumD*, Rif^R^	This Study
*ΔpgaA*	In frame deletion of *XOC_0767*, Rif^R^	This Study
*ΔpgaC*	In frame deletion of *XOC_0765*, Rif^R^	This Study
*ΔxagA*	In frame deletion of *XOC_3785*, Rif^R^	This Study
*ΔxagB*	In frame deletion of *XOC_3784*, Rif^R^	This Study
*ΔrpfG/gumD*	In frame deletion of *rpfG* and *gumD*, Rif^R^	This Study
*ΔrpfG/pgaA*	In frame deletion of *rpfG* and *XOC_0767*, Rif^R^	This Study
*ΔrpfG/pgaC*	In frame deletion of *rpfG* and *XOC_0765*, Rif^R^	This Study
*ΔrpfG/xagA*	In frame deletion of *rpfG* and *XOC_3785*, Rif^R^	This Study
*ΔrpfG/xagB*	In frame deletion of *rpfG* and *XOC_3784*, Rif^R^	This Study
**Plasmids**		
pUFR80	Suicide vector for homologous recombination, Km^R^	[60]
pVSP61	Expression vector, Km^R^	[61]
pMD18-T	High efficiency cloning vector, Amp^R^	Takara
pVSP61-*rpfGxoc*	Complementation, *rpfGxoc* cloned in pVSP61, Km^R^	This study
pVSP61-*rpfFxoc*	Complementation, *rpfFxoc* cloned in pVSP61, Km^R^	This study
pVSP61-*xagAxoc*	Complementation, *xagAxoc* cloned in pVSP61, Km^R^	This study
pQE30	*In-vitro* expression vector, Amp^R^	This study
pQE30-*rpfG*	*rpfG* cloned in pQE30 for RpfG purification, Amp^R^	This study

### Construction of *Xoc* Mutant Strains using Non-marker Homologous Recombination

Construction of *Xoc* mutant strains was performed following the procedures described by Sun *et al* with minor modifications [57], [58]. DNA was isolated from the *Xoc* wild-type strain RS105 using a genomic DNA isolation kit (New Industry Company, Beijing, China) following provided instructions. Two fragments approximately 800 bp to 1 kb long, upstream and downstream close to the start and stop codons of *rpfG*, were amplified separately via PCR from *Xoc* genomic DNA using *Pfu* polymerase. The used primer sets *rpfG*-*Xho*I-F/*rpfG*-del-R and *rpfG*-del-F/*rpfG*-*Hin*dIII-R are listed in Table S1 with underlined *Xho*I and *Hin*dIII restriction sites, respectively. PCR products were gel purified and added together into a fusion PCR reaction. The resultant PCR fragment carrying flanking regions of the *rpfG* gene but lacking the *rpfG* open reading frame was cloned into the pUFR80 *sacB* suicide vector [59], [60]. The pUFR80-*ΔrpfG* plasmid was transferred into *Xoc* RS105 by triparental mating and subjected to kanamycin selection. Single transformation colonies of *Xoc* with kanamycin resistance were picked and cultured overnight in NB medium without kanamycin and sucrose, then spread onto NA plates with 5% sucrose to screen sucrose-insensitive clones. The gene-deletion genotype of kanamycin-sensitive/sucrose-insensitive *Xoc* colonies was confirmed by colony PCR and sequencing PCR products, as well as by Southern blot analyses. The same strategy was applied to construct other gene-deletion strains including *ΔhgdA*, *ΔhgdC*, *ΔgumD*, *ΔpgaA*, *ΔpgaC*, *ΔxagA* and *ΔxagB* except that different restriction enzyme sites were created by PCR for deletion fragments (Table S1). Single and double unmarked mutants were used to construct the second and third gene deletion, respectively.

### Construction of Complementation Strains for *Xoc* Mutant Strains

For complementation, the full-length *rpfG* gene including the 5′- and 3′- regulatory sequences (687 bp and 225 bp respectively) were amplified by PCR using the respective primer sets *rpfG*-*Xho*I-F/*rpfG*-*Hin*dIII-R (Table S1). The resultant PCR fragments were cloned into the wide host range vector pVSP61 [61] and mated into the specified *Xoc* strains. Hence the *rpfG* complementation construct carried short segments of adjacent open reading frames from separate operons but no other full-length genes. All other complementation strains were constructed using the same procedure with the primer sets listed in Table S1. All constructs were subjected to sequencing.

### Southern Blot Analysis

Southern blot analysis was performed using standard molecular biology methods unless noted [62]. Briefly, genomic DNA was isolated from *Xoc* strains as described above and then digested with appropriate restriction enzymes. After separated with agarose gel, genomic DNA was blotted onto nylon membrane and probed with a ^32^P-labeled PCR product generated with the primer sets *rpfG*-probe-F/*rpfG*-probe-R, *hgdA*-probe-F/*hgdA*-probe-R and *hgdC*-probe-F/*hgdC*-probe-R, respectively (Table S1).

### Site-directed Mutagenesis

Site-directed mutagenesis for changing HD residues to AA residues in HD-GYP domain proteins was performed by two-step fusion PCR [57]. In the first round of PCR, two separate reactions were carried out using the primer sets, *rpfG*-*Xho*I-F/*rpfG*-MutHD-R and *rpfG*-MutHD-F/*rpfG*-*Hin*dIII-R, respectively. The primers *rpfG*-MutHD-R and *rpfG*-MutHD-F were intentionally designed to be partially complementary to each other and to change the His-Asp codons to Ala-Ala codons (Table S1). DNA fragments amplified from the first round of PCR were added together in a fusion PCR with the primer set *rpfG*-*Xho*I-F and *rpfG*-*Hin*dIII-R. The resultant PCR products were then subcloned into the pVSP61 expression vector for functional studies.

### Biofilm Assays

The protocol for measuring biofilm formation was adapted from the method described by O’Toole and Kolter [63]. Briefly, overnight bacterial cultures were inoculated into 5 ml L medium (tryptone, 10 g/L; yeast extract, 5 g/L; NaCl, 5 g/L; glucose, 1 g/L) with 1∶1000 dilution and incubated in the borosilicate glass tubes without shaking at 28°C for 1 week. The cultured cells were then stained with crystal violet (CV) for 15 min. The unbound dye was removed by rinsing with H_2_O. The glass-bound dye was solubilized in 90% ethanol and quantified by spectrophotometry at 590 nm.

### Quantitative Determination of EPS

The quantity of EPS produced in *Xoc* strains was determined using the method as described [64], [65]. Briefly, overnight cultures of the *Xoc* wild-type and mutant strains were collected and re-suspended in sterile water to an OD600 of 1.0. The cells were then diluted at 1∶1000 in M210 medium (casein enzymatic hydrolysates, 8 g/L; yeast extract, 4 g/L; sucrose, 5 g/L; KH_2_PO_4_, 3 g/L; MgSO_4_.7H_2_O, 0.3 g/L) and cultured overnight to cell density of OD600≈2. The cell cultures (10 ml) were collected by centrifugation at 12,000 rpm for 10 min. The supernatants were mixed with two volumes of absolute ethanol and incubated at –20°C overnight to precipitate EPS. The pellet was then collected by centrifugation at 10,000 rpm for 5 min and fully dried at 55°C before weighing.

### Protease Assays

The secretion of proteases in *Xoc* strains was evaluated on the plates with skimmed milk [66]. Overnight cultures of *Xoc* were collected by centrifugation and re-suspended in sterile water to cell density of 10^9^ cfu/ml. Five microliter of cells were spotted onto nutrient agar (NYGA) or water agar plates containing 1% (w/v) skimmed milk and incubated at 28°C for 4 days. The proteolytic activity of *Xoc* strains was quantified by measuring the diameter of clearing zones around the colonies that were formed after proteolytic degradation of milk proteins.

### Motility Assays

Swimming motility of *Xoc* strains was investigated on semisolid medium plates with 0.3% noble agar as described by DiLuzio *et al* [67]. All *Xoc* strains were inoculated into the center of the plates by pipetting. After incubating at 28°C for 4 days, the colony diameter was measured.

### Virulence Assays of *Xoc* Strains on Rice

Virulence on rice of different *Xoc* strains was investigated by pressure inoculation [5]. Overnight *Xoc* cultures were diluted to an OD600 of 0.3 and injected into the leaves of 6-week-old rice plants with needleless syringes. The length of disease lesion on the leaves was measured at 14 to 20 days after inoculation. At least 10 leaves were inoculated and scored for each tested *Xoc* strain. For establishing growth curves, inoculated rice leaves were harvested at four time points (0, 5, 10, 15 days after inoculation), immediately sliced into small pieces, incubated in 1 ml sterile water including 25 µg/ml of rifampicin with shaking for 1 h, and then filtered through two layers of sterilization gauze. The filtrates were diluted and then plated onto NA agar plates with antibiotics. Colonies on the plates were counted after 3 days of incubation at 28°C [68].

### *In vitro* Protein Expression and Purification

The open reading frame of *rpfG* was amplified from *Xoc* RS105 genome by PCR using primers *rpfG*-*Bam*HI-F and *rpfG*-*Hin*dIII-R (seen in Table S1). The PCR fragment was subcloned into the pQE30 expression vector (Qiagen) after digestion with *Bam*HI and *Hin*dIII. The construct was transformed into *E. coli* XL1-blue cells and sequenced to confirm no nucleotide changes. Cells were grown in 5 ml of LB medium containing ampicillin overnight at 37°C. The culture was 1∶50 diluted and grown further until it reached to an OD600 of 0.5∼0.7. Isopropyl β-D-thiogalactopyranoside (IPTG) was then added to a final concentration of 1 mM to induce the expression of proteins at 28°C. After 3 h of incubation, the cells were collected by centrifugation, re-suspended in lysis buffer (50 mM NaH_2_PO_4_, 300 mM NaCl, and 10 mM imidazole, pH 8.0), and then sonicated with 10 s pauses at 200∼300 W for 6 times. The lysates were centrifuged at 10,000 g for 30 min and the supernatant was then loaded onto nickel-nitrilotriacetic acid agarose superflow columns (Qiagen), which were subsequently rinsed with wash buffer (50 mM NaH_2_PO_4_, 300 mM NaCl, and 20 mM imidazole, pH 8.0). The bound His6-tagged proteins were eluted with elution buffer (50 mM NaH_2_PO_4_, 300 mM NaCl, and 250 mM imidazole, pH 8.0) and dialyzed extensively in PBS (pH 7.4). The concentration of proteins was determined using the BCA protein assay kit (Pierce).

### PDE Colorimetric Assays

The PDE activity of *in vitro* purified proteins were assayed by incubation with bis(p-nitrophenyl) phosphate [69]. Purified proteins (20 µg) were incubated with 5 mM bis(p-nitrophenyl) phosphate at 37°C for 1.5 h in assay buffers (50 mM Tris-HCl, 1 mM MnCl_2_, pH 8.5). The release of p-nitrophenol was then quantified at OD_410_ using spectrophotometer.

### PDE Enzyme Assay by HPLC and Mass Spectrometry

The PDE activity of purified proteins was also assayed by detecting the degradation of c-di-GMP as described [7], [8]. The reaction assay mix included 20 µg purified protein, 100 µM c-di-GMP, 50 mM Tris-HCl (pH 7.6), 10 mM MgCl_2_, 10 mM MnCl_2_, 0.5 mM EDTA and 50 mM NaCl in a total volume of 600 µl. After incubated in 37°C for 6 h, the reaction mix was boiled for 3 min to stop the reaction. The supernatant was collected by centrifugation at 15,000 g for 2 min and filtered through a 0.22 µm filter. The HPLC analysis was performed on a reversed phase C18 column (250×4.60 mm; Phenomenex, USA) with an Agilent 1100 series. The samples were separated at a flow rate of 1 ml/min under isocratic condition in eluent A (20 mM potassium phosphate buffer, pH 5.8, containing 1% methanol) at the first three minutes and then on a linear gradient from 0–20% methanol in the next 20 minutes.

Separation of nucleotides for mass spectrometry was performed on a reversed phase C18 column in a liner gradient from 0–20% buffer B (acetonitrile containing 0.01% formic acid) in buffer A (0.1% ammonium formate, pH 3.7). Mass spectrometry was operated at negative ion mode with Agilent 1100 series LC/MSD Trap (VL). The GMP and c-di-GMP standards were purchased from Sigma (USA) and Biolog (Germany), respectively.

### RNA Isolation and qRT-PCR

Overnight *Xoc* cultures were diluted in XOM3 medium to an OD600 of 0.08 and grown till OD600 = 0.6, and then harvested for RNA isolation. RNA was isolated using PureYield™ RNA midiprep System (Promega) according to the manufacturer’s instructions. The isolated RNA was used as a template in a PCR reaction with the primer set 16S-RNA-F/16S-RNA-R (seen in Table S1) to confirm no DNA contamination. RNA (60 ng) was then used to synthesize cDNA with the TransScript II first-strand cDNA synthesis supermix (Transgen, Beijing, China). The SYBR Green premix ExTaq (Takara) was used in qRT-PCR reactions to quantify the transcript levels. 16S rRNA was used as the internal reference for data analysis.

### Promoter-GUS Fusion

The promoter regions of *hrpG*, *hrpX* and *hrpA* were amplified from *Xoc* RS105 genome by PCR using primers *hrpG*-pro-F/*hrpG*-pro-R, *hrpX*-pro-F/*hrpX*-pro-R and *hrpA*-pro-F/*hrpA*-pro-R, respectively (Table S1). PCR products were subcloned into the pJY-Tn7T-GUS vector after digestion with appropriate restriction enzymes [70]. After verified by sequencing, constructed plasmids and the transposase pTNS-1 helper plasmid were co-transferred into *Xoc* strains by parental mating [71]. The insertion-containing transformants were screened on the NA plates supplemented with 25 µg/ml rifampicin, 50 µg/ml streptomycin, 100 µg/ml spectinomycin. Colony PCR with primers GUS-1/GUS-2 (Table S1) was used to confirm the promoter-GUS fusion.

Bacterial cells were collected by centrifugation at 12,000 rpm for 3 min. Total soluble proteins were prepared by sonication in lysis buffer (20 mM Tris-HCl, pH 7.4; 5 mM EDTA, 10 mM mercaptoethanol, 1% Triton X-100). The GUS activity of soluble proteins was assessed using 4-methylumbelliferyl-β-D-glucuronide(MUG) as substrates according to the standard protocol [72], [73]. Fluorescence was measured on an Infinite F200 microplate reader (TECAN, Austria) with excitation at 360 nm and emission at 485 nm.

### Statistical Analysis

Means and standard errors of experimental data were calculated using Microsoft Office Excel. All statistical analyses were performed by Duncan’s multiple range test using SAS software. P<0.05 was considered statistically significant.

## Supporting Information

Figure S1 **Predicted domain organizations of three HD-GYP domain proteins HgdA (XOC1984), RpfG (XOC2264) and HgdC (XOC4564).** HgdA and RpfG have an HD-GYP domain in association with an N-terminal CheY-like response receiver (REC) regulatory domain. HgdC has an HD-GYP domain with additional, uncharacterized N-terminal and C-terminal domains. The numbers indicate amino acid residue positions.
(TIF)

Figure S2 ***Xoc rpfG*** **-related mutants were verified by Southern blot analyses.** Digested genomic DNA was separated, blotted onto membrane and then probed with the isotope-labelled *rpfG*-probe (A), *hgdA*-probe (B) and *hgdC*-probe (C) PCR fragments. A, Genome DNA from the wild-type (lane 1 and 2) and *ΔrpfG* (lane 3 and 4) strains digested by *Apa*I (lane 1 and lane3) and *Bam*HI (lane 2 and 4) was hybridized with *rpfG*-probe. B, Genome DNA from the indicated mutant strains digested by *Kpn*I and *Eco*RI/*Eco*RV was hybridized with *hgdA*-probe. C, Genome DNA from *hgdC*-related mutant strains digested by *Bam*HI and *Sma*I was hybridized with *hgdC*-probe. The primers were designed to amplify DNA fragments as probes that do not hybridize with genome DNA of mutant strains because the fragments were deleted via homologous recombination. M: Marker.
(TIF)

Figure S3 **The effect of** ***rpfF*** **mutation on biofilm formation in** ***Xoc*** **.** Biofilm formation was dramatically increased in *Xoc ΔrpfF* mutant when cultured in L-medium. Complementation with introduction of the full-length *rpfF* gene to produce the *ΔrpfF(rpfF)* strain reduced biofilm formation to the wild-type level. WT: wild-type.
(TIF)

Figure S4 **Effects of** ***hgdA*** **,** ***rpfG*** **and** ***hgdC*** **mutations on swimming motility and protease secretion in** ***Xoc*** **.** (A) The amount of secreted proteases in *Xoc* was assessed by the diameter of clearing zones produced after the hydrolysis of skimmed milk on water agar plates. (B) Swimming motility of the *Xoc* wild-type and mutant strains was determined on semisolid plates with 0.3% noble agar. The motility was indicated by the diameter (cm) of the radial growth.
(TIF)

Figure S5 **The phosphodiesterase activity of** ***Xoc*** **RpfG.** ***Xoc*** **RpfG was** ***in vitro*** **expressed as an N-terminal His6-tagged fusion and then purified using nickel columns under native conditions.** The PDE activity of *Xoc* RpfG against c-di-GMP was assessed by reverse phase High Performance Liquid Chromatography (HPLC). (A-D) HPLC analyses of the RpfG*_Xoc_* PDE activity using c-di-GMP as a substrate. (A) and (B) GMP and c-di-GMP standard. (C) The purified RpfG*_Xoc_* had activity against standard cyclic di-GMP, generating two hydrolytic products with the retention time at 6.024 s and 12.922 s, respectively after purified RpfG*_Xoc_* was incubated with c-di-GMP for 6 h. (D) Reaction control without RpfG*_Xoc_*. C-di-GMP was stable and no degraded product but only c-di-GMP was detected. (E–F) Mass spectrometry, operated at negative ion mode, was used to confirm the identity of HPLC fractions in Figure S5C. (E) The GMP peak was detected by LC-MS at an *m/z* of 362.0. (F) The second peak was distinct from c-di-GMP and GMP with a [M-H]^+^ *m/z* at 707.1, which corresponds to the intermediate product pGpG.
(TIF)

Figure S6 **The growth rate of** ***ΔrpfG*** **compared to those of the wild-type and complemented strains in XOM3 minimal medium.** The bacterial population was determined by counting colony forming units after manual plating at the indicated time points. WT: wild-type.
(TIF)

Figure S7 **Effects of** ***xagA*** **,** ***xagB*** **,** ***pgaA*** **,** ***pgaC*** **and** ***gumD*** **deletions on swimming motility in** ***Xoc*** **.** Swimming motility of the *Xoc* wild-type, mutant and complementation strains was determined on semisolid plates with 0.3% noble agar. The motility was indicated by the diameter (cm) of the radial growth.
(TIF)

Table S1 **Primers used in this study.**
(DOC)
