# Supplementary figures and images for: The HD-GYP Domain Protein RpfG of Xanthomonas oryzae pv. oryzicola Regulates Synthesis of Extracellular Polysaccharides that Contribute to Biofilm Formation and Virulence on Rice
Source: PLoS One. 2013 Mar 27;8(3):e59428. doi: 10.1371/journal.pone.0059428 (PMC3609779; doi:10.1371/journal.pone.0059428)

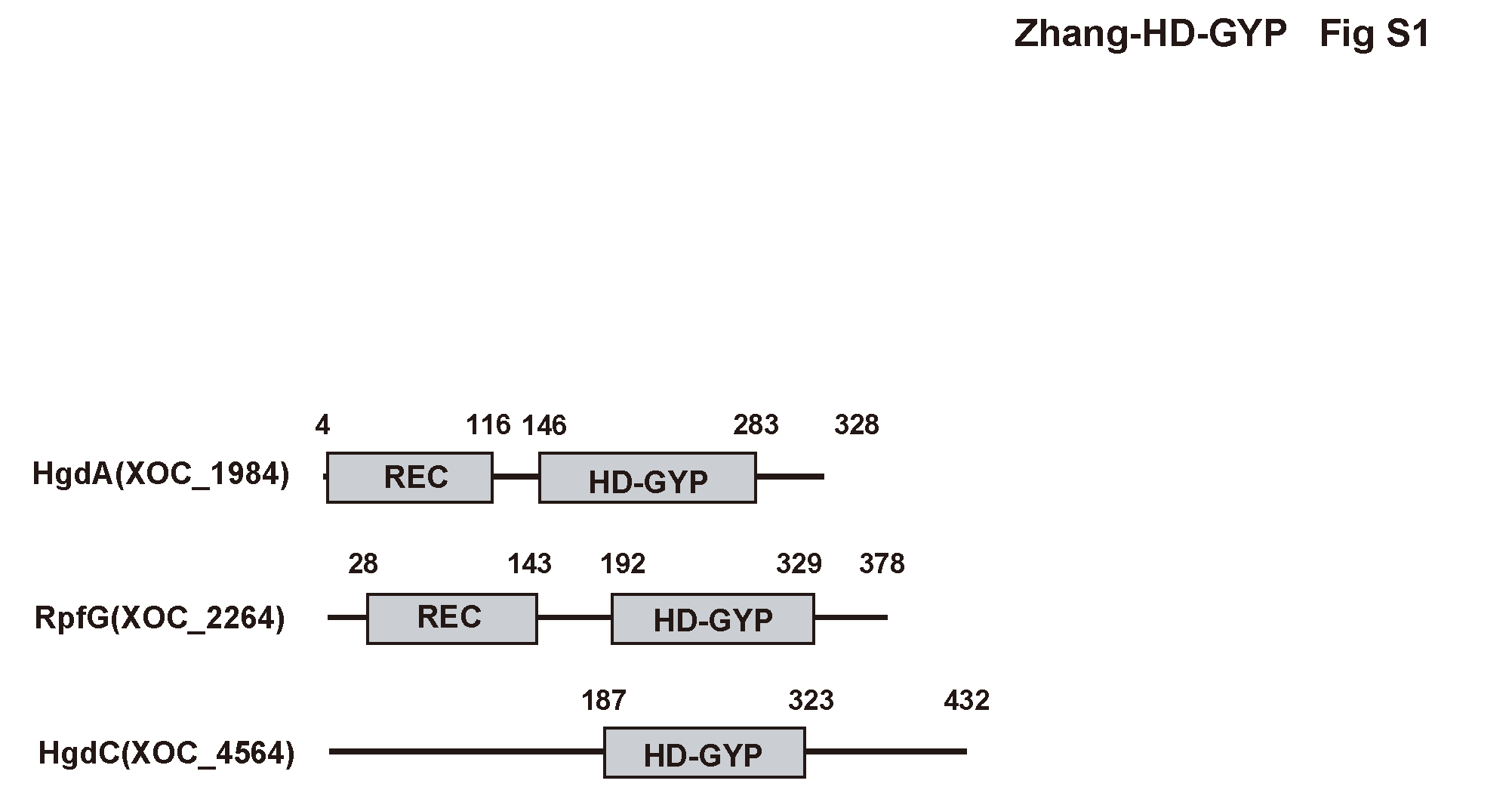

Supplement: Figure S1 — Predicted domain organizations of three HD-GYP domain proteins HgdA (XOC1984), RpfG (XOC2264) and HgdC (XOC4564). HgdA and RpfG have an HD-GYP domain in association with an N-terminal CheY-like response receiver (REC) regulatory domain. HgdC has an HD-GYP domain with additional, uncharacterized N-terminal and C-terminal domains. The numbers indicate amino acid residue positions. (TIF) [file pone.0059428.s001.tif]

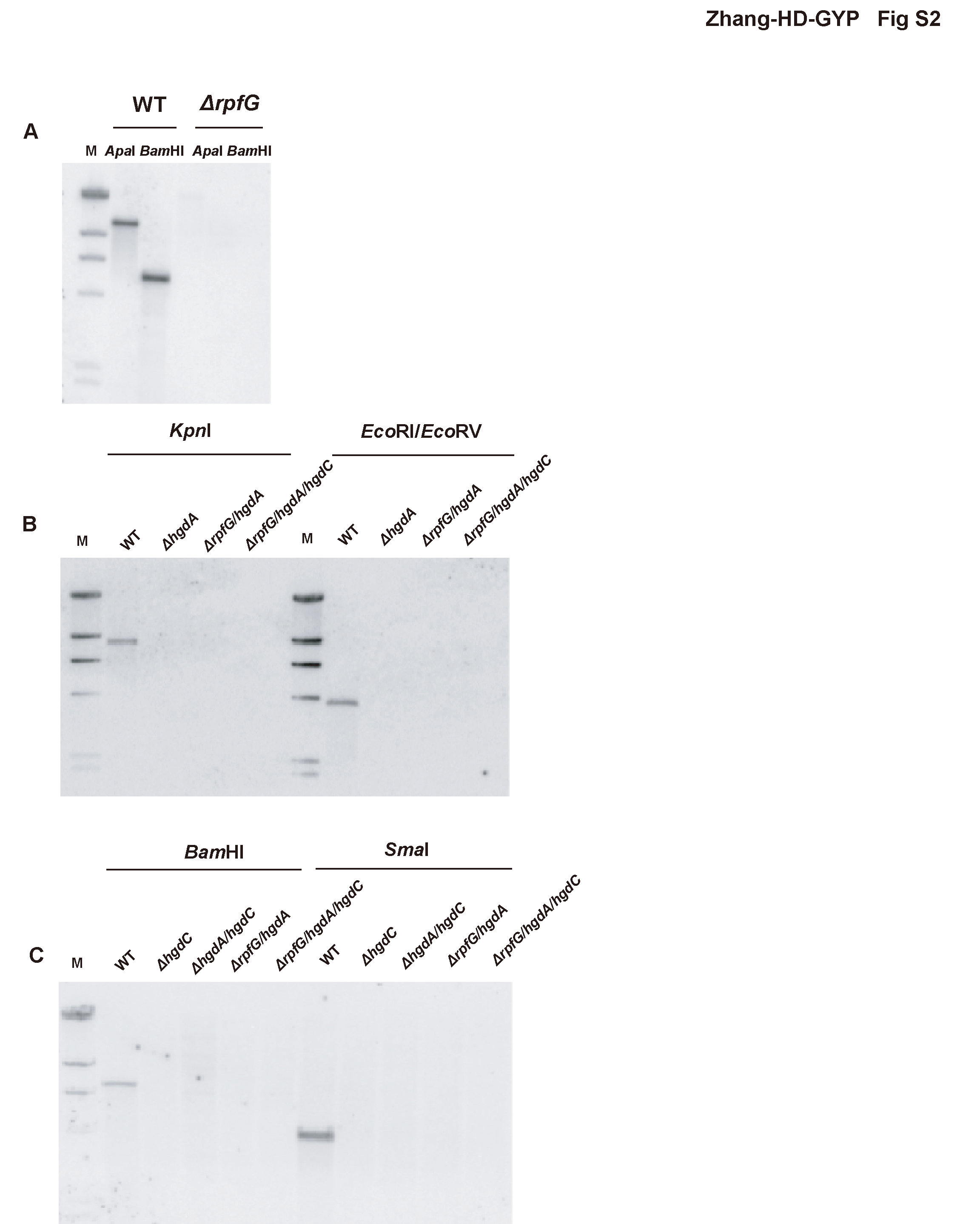

Supplement: Figure S2 — Xoc rpfG -related mutants were verified by Southern blot analyses. Digested genomic DNA was separated, blotted onto membrane and then probed with the isotope-labelled rpfG-probe (A), hgdA-probe (B) and hgdC-probe (C) PCR fragments. A, Genome DNA from the wild-type (lane 1 and 2) and ΔrpfG (lane 3 and 4) strains digested by ApaI (lane 1 and lane3) and BamHI (lane 2 and 4) was hybridized with rpfG-probe. B, Genome DNA from the indicated mutant strains digested by KpnI and EcoRI/EcoRV was hybridized with hgdA-probe. C, Genome DNA from hgdC-related mutant strains digested by BamHI and SmaI was hybridized with hgdC-probe. The primers were designed to amplify DNA fragments as probes that do not hybridize with genome DNA of mutant strains because the fragments were deleted via homologous recombination. M: Marker. (TIF) [file pone.0059428.s002.tif]

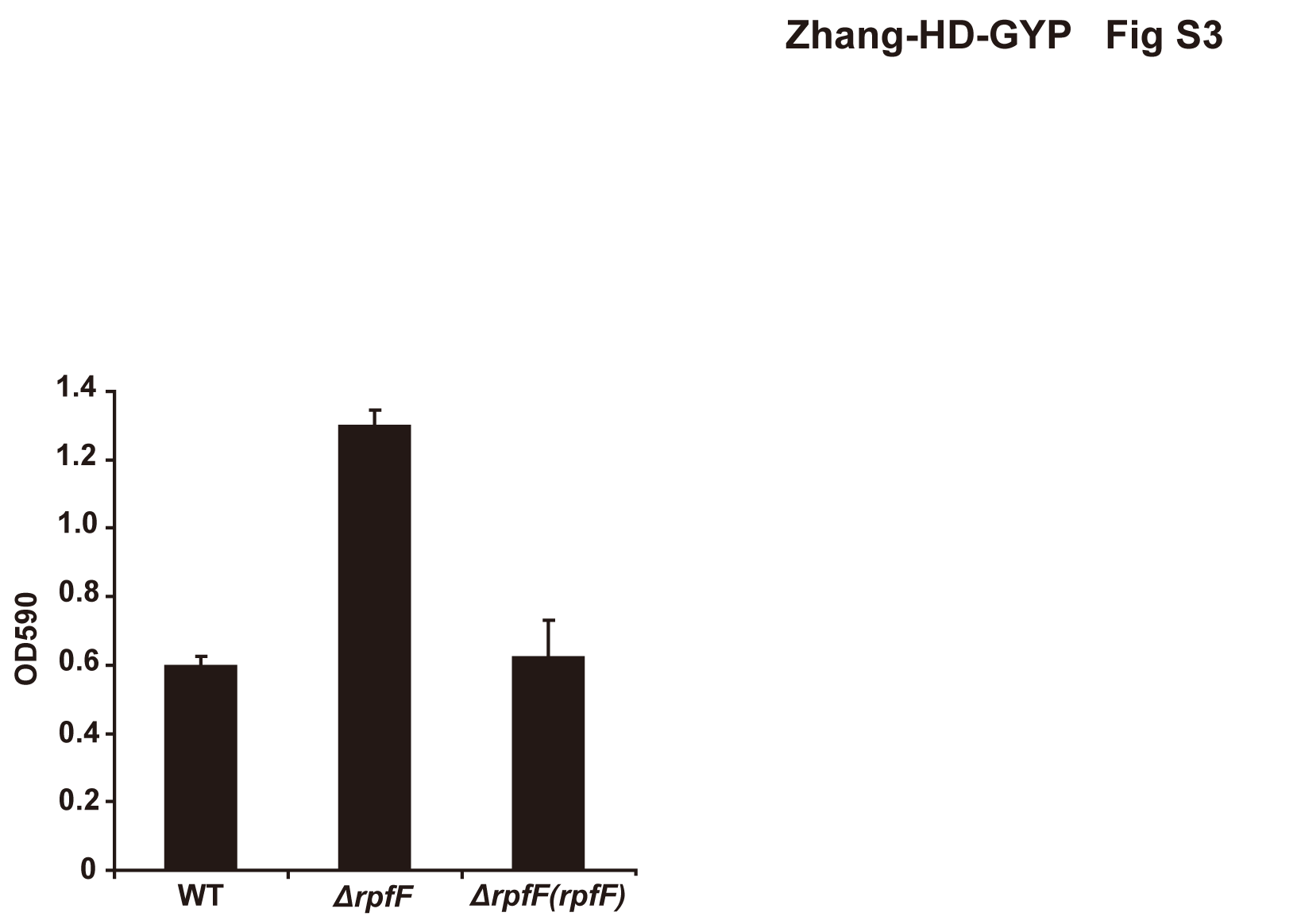

Supplement: Figure S3 — The effect of rpfF mutation on biofilm formation in Xoc . Biofilm formation was dramatically increased in Xoc ΔrpfF mutant when cultured in L-medium. Complementation with introduction of the full-length rpfF gene to produce the ΔrpfF(rpfF) strain reduced biofilm formation to the wild-type level. WT: wild-type. (TIF) [file pone.0059428.s003.tif]

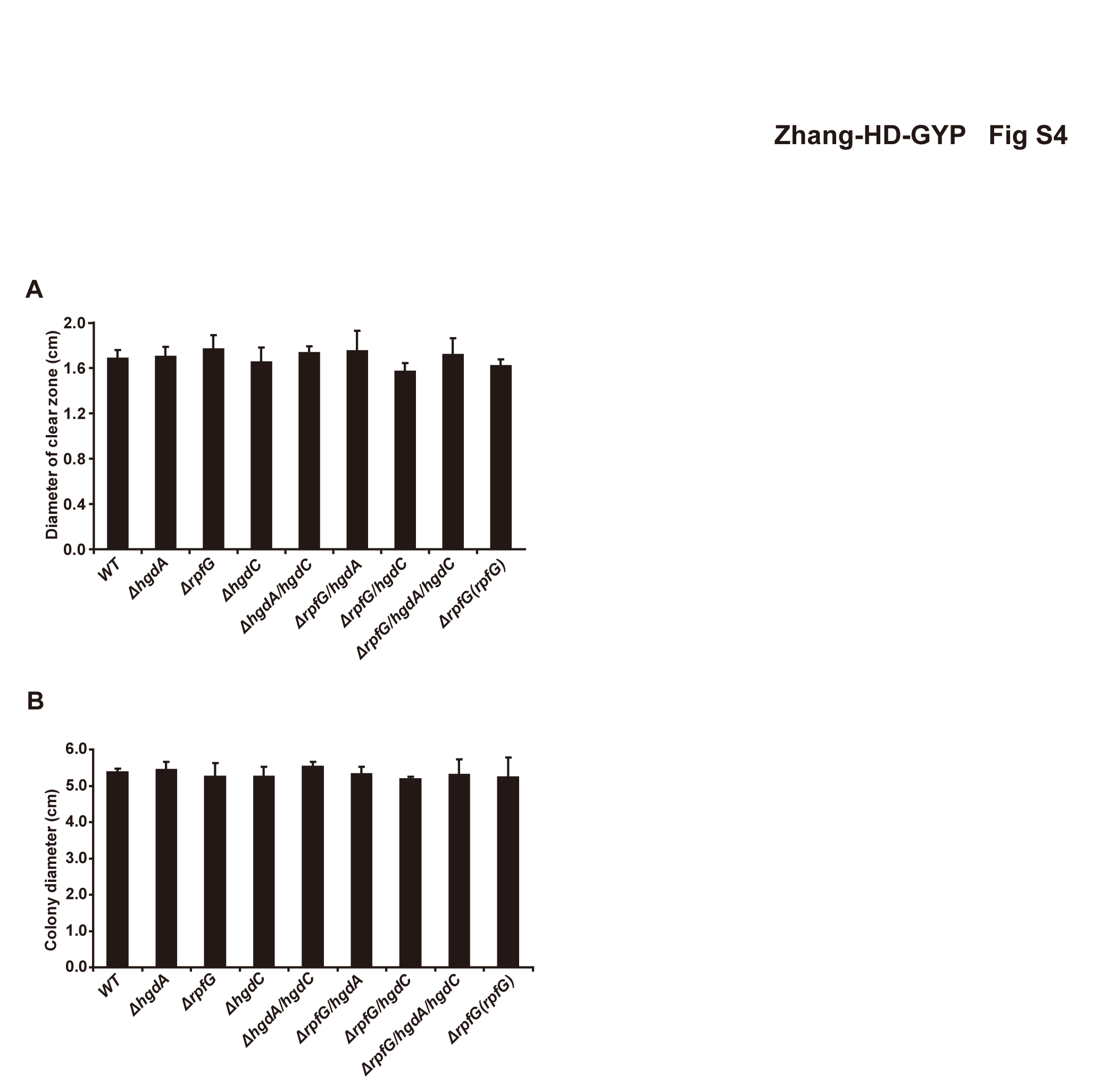

Supplement: Figure S4 — Effects of hgdA , rpfG and hgdC mutations on swimming motility and protease secretion in Xoc . (A) The amount of secreted proteases in Xoc was assessed by the diameter of clearing zones produced after the hydrolysis of skimmed milk on water agar plates. (B) Swimming motility of the Xoc wild-type and mutant strains was determined on semisolid plates with 0.3% noble agar. The motility was indicated by the diameter (cm) of the radial growth. (TIF) [file pone.0059428.s004.tif]

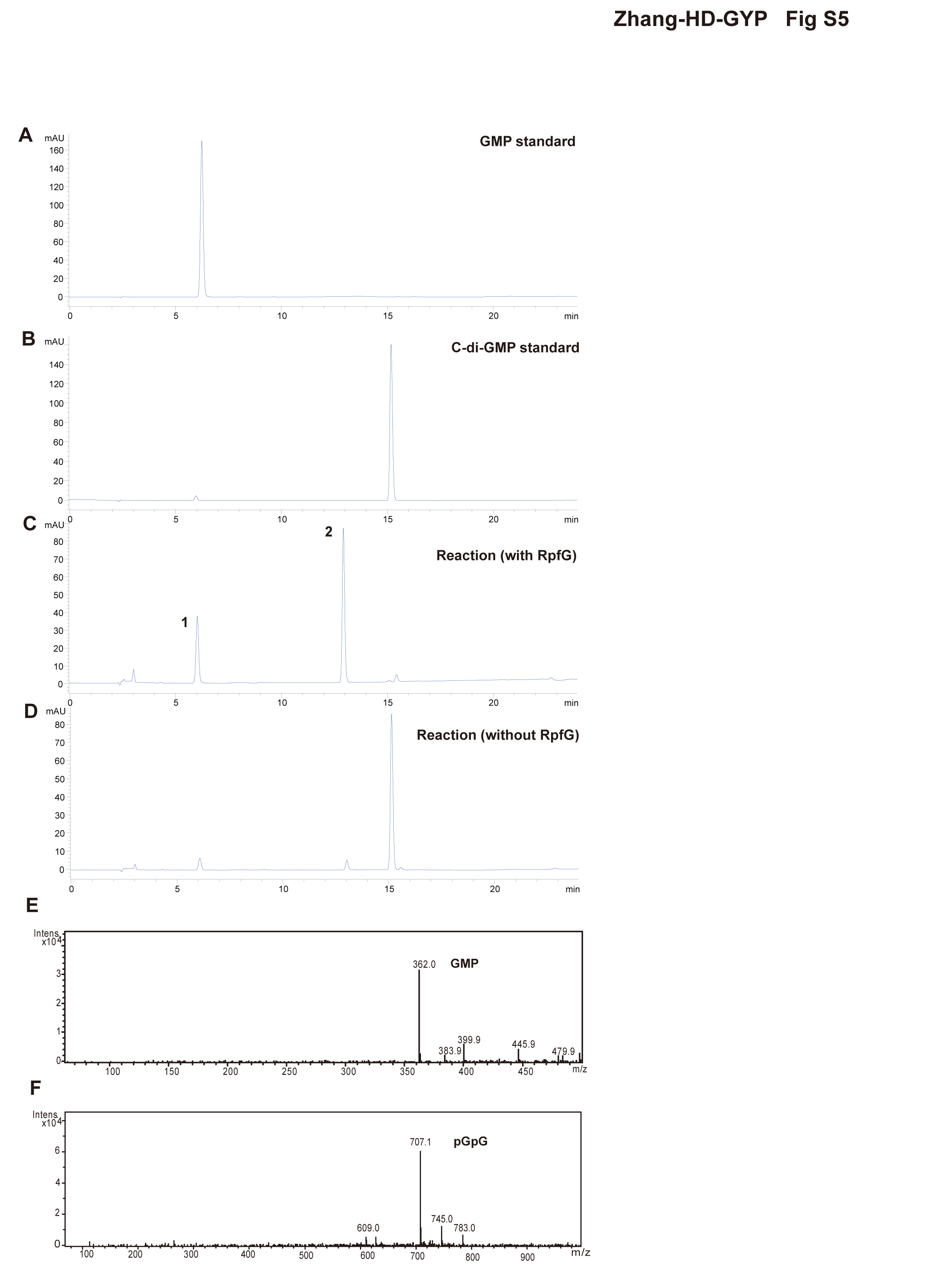

Supplement: Figure S5 — The phosphodiesterase activity of Xoc RpfG. Xoc RpfG was in vitro expressed as an N-terminal His6-tagged fusion and then purified using nickel columns under native conditions. The PDE activity of Xoc RpfG against c-di-GMP was assessed by reverse phase High Performance Liquid Chromatography (HPLC). (A-D) HPLC analyses of the RpfGXoc PDE activity using c-di-GMP as a substrate. (A) and (B) GMP and c-di-GMP standard. (C) The purified RpfGXoc had activity against standard cyclic di-GMP, generating two hydrolytic products with the retention time at 6.024 s and 12.922 s, respectively after purified RpfGXoc was incubated with c-di-GMP for 6 h. (D) Reaction control without RpfGXoc. C-di-GMP was stable and no degraded product but only c-di-GMP was detected. (E–F) Mass spectrometry, operated at negative ion mode, was used to confirm the identity of HPLC fractions in Figure S5C. (E) The GMP peak was detected by LC-MS at an m/z of 362.0. (F) The second peak was distinct from c-di-GMP and GMP with a [M-H]+ m/z at 707.1, which corresponds to the intermediate product pGpG. (TIF) [file pone.0059428.s005.tif]

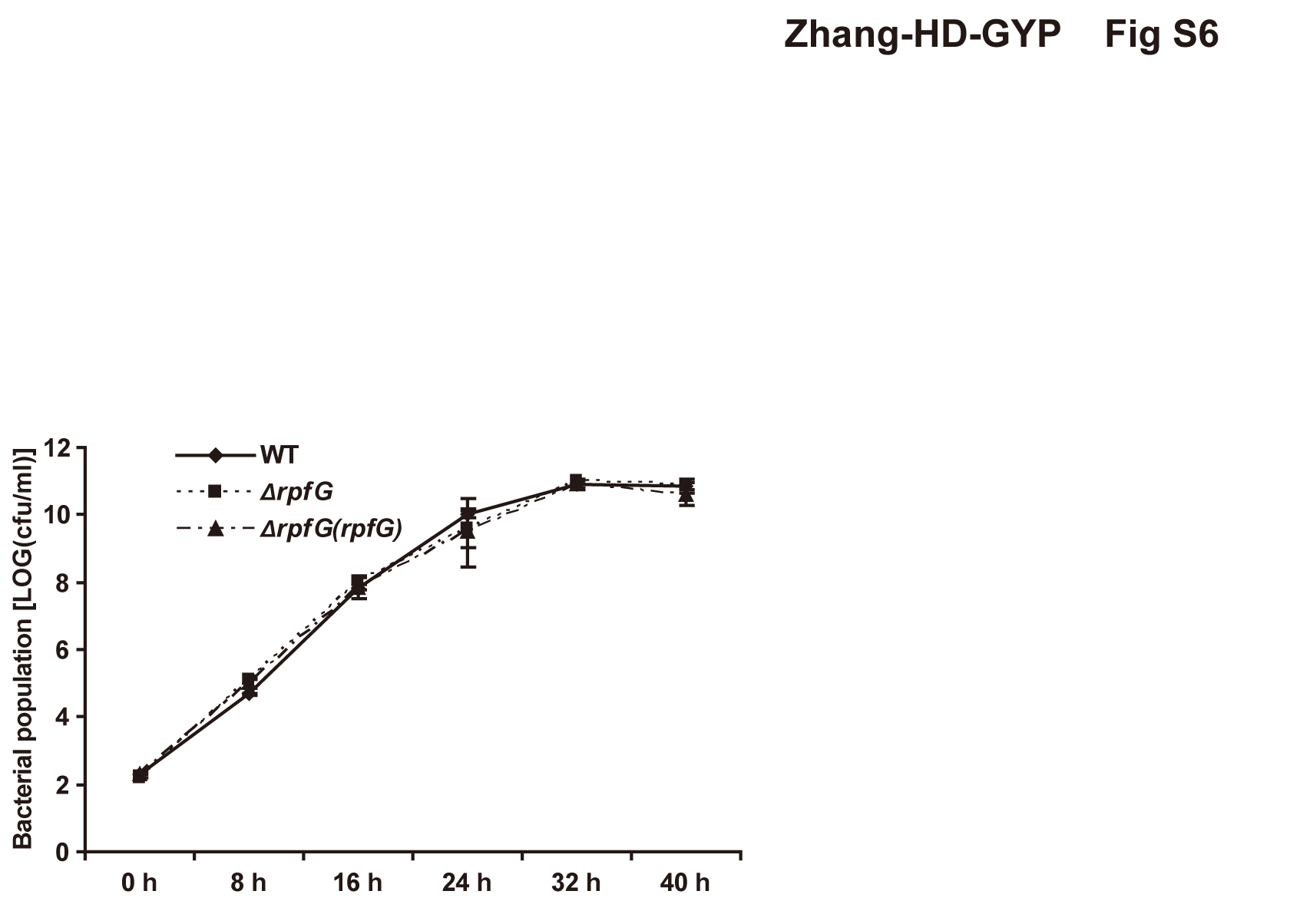

Supplement: Figure S6 — The growth rate of ΔrpfG compared to those of the wild-type and complemented strains in XOM3 minimal medium. The bacterial population was determined by counting colony forming units after manual plating at the indicated time points. WT: wild-type. (TIF) [file pone.0059428.s006.tif]

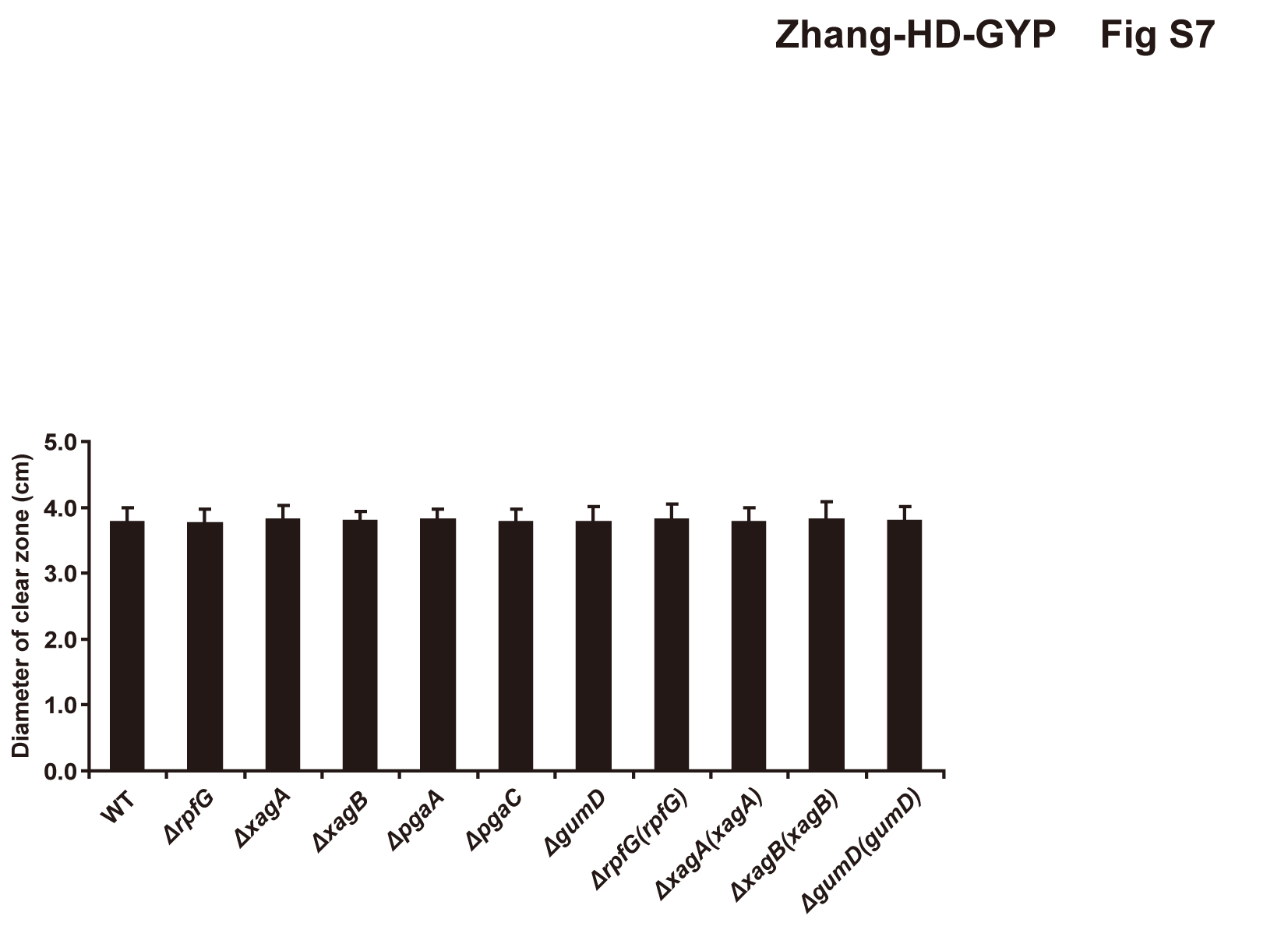

Supplement: Figure S7 — Effects of xagA , xagB , pgaA , pgaC and gumD deletions on swimming motility in Xoc . Swimming motility of the Xoc wild-type, mutant and complementation strains was determined on semisolid plates with 0.3% noble agar. The motility was indicated by the diameter (cm) of the radial growth. (TIF) [file pone.0059428.s007.tif]
