# Supplementary material for: The HD-GYP Domain Protein RpfG of Xanthomonas oryzae pv. oryzicola Regulates Synthesis of Extracellular Polysaccharides that Contribute to Biofilm Formation and Virulence on Rice
Source: PLoS One. 2013 Mar 27;8(3):e59428. doi: 10.1371/journal.pone.0059428 (PMC3609779; doi:10.1371/journal.pone.0059428)
Supplement: Table S1 — Primers used in this study. (DOC) [file pone.0059428.s008.doc]

**Table S1.** Primers used in this study^a^

| *Gene* | Primer name | DNA sequence |
| --- | --- | --- |
| *rpfG* | *rpfG*-*Xho*I-F | GCTCGAGCTCTACGAGGCCTATG |
|  | *rpfG*-del-R | CCGATGCGGTGGAGAATTCGTGCAGTATGTGTGCCGTCGG |
|  | *rpfG*-del-F | CCGACGGCACACATACTGCACGAATTCTCCACCGCATCGG |
|  | *rpfG*-*Hin*dIII-R | TTAAGCTTGAATCGAAGTACAGCGGCACC |
|  | *rpfG*-MutHD-R | TTGCCCATGGCAGCCAGCAGCCCGCCCATTGCCG |
|  | *rpfG*-MutHD-F | ACCGCTGGCAGCAATGGGCAAGATCGCCATTCCC |
|  | *rpfG*-*Bam*HI-His-F | AAGGATCCGATGTTTCAGGAGGTCCGGG |
|  | *rpfG*-*Hin*dIII-His-R | TTAAGCTTATCACGCAGCTGACCAGACG |
|  | *rpfG*-probe-F | TTTCCGTTCCCGAGAATAGAGGAGC |
|  | *rpfG*-probe-R | CGGGAATGGCGATCTTGCCC |
| *hgdA* | *hgdA*-*Eco*RI-F | AGAATTCCGTCGCTTCCGCATGAGA |
|  | *hgdA*-del-R | CTAGATCACGCTATGGCGCAGGTGGATCCTTGCCAAGGCT |
|  | *hgdA*-del-F | AGCCTTGGCAAGGATCCACCTGCGCCATAGCGTGATCTAG |
|  | *hgdA*-*Hin*dIII-R | ATAAGCTTCAAGGCAATGGCCGCAACG |
|  | *hgdA*-probe-F | CTGGTGTTCGCCAAATCCG |
|  | *hgdA*-probe-R | CCGTCCCAGCATTCGTGAT |
| *hgdC* | *hgdC*-*Eco*RI-F | AGAATTCAACGCGGTATTGGCACGGAT |
|  | *hgdC*-del-R | CCGCTTACGGCAGCCAGAGTATGCCGGCTCTGCTGTGCAC |
|  | *hgdC*-del-F | GTGCACAGCAGAGCCGGCATACTCTGGCTGCCGTAAGCGG |
|  | *hgdC*-*Hin*dIII-R | TTAAGCTTGAGGTCGAACGCTGCCAC |
|  | *hgdC*-probe-F | ACCGCCGACGACTACACCT |
|  | *hgdC*-probe-R | CAACGAATCGGGCGTCTGT |
| *rpfF* | *rpfF*-*Xho*I-F | AAACTCGAGCTGAAAAAGACCCGCTTCAC |
|  | *rpfF*-del-R | AGCGAATAGCCCGCCGCACACGTCGTCGGAGACTGAACGT |
|  | *rpfF*-del-F | ACGTTCAGTCTCCGACGACGTGTGCGGCGGGCTATTCGCT |
|  | *rpfF*-*Hin*dIII-R | TTTAAGCTTCTGGTTCGAGTTGCAGCTGG |
| *hrpG* | *hrpG*-pro-F | TTGAATTCGCGTAAATCGCTGGGCTCG |
|  | *hrpG*-pro-R | TATAAGCTTCAGAAACGCCGATCCGTGTGC |
|  | *hrpG*-qRT-F | GAGTTACTGGTCTTCGACGC |
|  | *hrpG*-qRT-R | CCAGTCCAGGATGTCATTGG |
| *hrpX* | *hrpX* -pro-F | TATAAGCTTGGCTGAGACTTGGGCTGAC |
|  | *hrpX* -pro-R | TAGGTACCTGAAAGAGACCGAAAAAATCAAAAAATCTC |
|  | *hrpX*-qRT-F | TCCTTTCGACCTACTTTGCAG |
|  | *hrpX*-qRT-R | TCGCTGTTGAAGGTGCTG |
| *hrpA* | *hrpA* -pro-F | TATAAGCTTATGCGGCTGCGTTCCAGC |
|  | *hrpA* -pro-R | TATCTAGATGATCGAAAAATGTGAAAGATTGAATGACG |
|  | *hrpA*-qRT-F | ATCAAGTTCATGACCGACGG |
|  | *hrpA*-qRT-R | ACTGTTTGAGATAGCCCAGC |
| *gumD* | *gumD*-*Eco*RI-F | AAGAATTCGAAGAGTCGCGTCGGCAGATC |
|  | *gumD*-del-R | TAGTGACGCTGCATGTAGTGGTCAGGTCGAAGACGCGCA |
|  | *gumD*-del-F | TGCGCGTCTTCGACCTGACCACTACATGCAGCGTCACTA |
|  | *gumD*-*Hin*dIII-R | TTAAGCTTGGGGTTGATCAGATCGCCGTA |
| *pgaA* | *pgaA*-qRT-F | TGTTTGACCAGGACCAGAAG |
|  | *pgaA*-qRT-R | TGTCTTCGCTTTCGCCATAG |
|  | *pgaA*-*Eco*RI-F | AAGAATTCAGGCCGATGGACGATACGCAG |
|  | *pgaA*-del-R | CACCGTATTCACATGTCCGGCTTGAGGTGGCTGCATGTCC |
|  | *pgaA*-del-F | GGACATGCAGCCACCTCAAGCCGGACATGTGAATACGGTG |
|  | *pgaA*-*Hin*dIII-R | TAAAGCTTACCGTACGGATTGGACTGCACC |
| *pgaB* | *pgaB*-qRT-F | ACCGAATTACCGCAGTTACC |
|  | *pgaB*-qRT-R | CGGTTGTGCGTAAAGATTGC |
| *pgaC* | *pgaC*-qRT-F | ATGCCGGAAACCCTCAAG |
|  | *pgaC*-qRT-R | CAGGACGCTCAGGATGTATTC |
|  | *pgaC*-*Bam*HI-F | AAGGATCCGGACGGTAACAACACCGCCAG |
|  | *pgaC*-del-R | CACAGGCTGAGCAACCAGTATGTCACTTGTCCGGGTAAGGG |
|  | *pgaC*-del-F | CCCTTACCCGGACAAGTGACATACTGGTTGCTCAGCCTGTG |
|  | *pgaC*-*Hin*dIII-R | TTAAGCTTCCTTGCTGCGGCGCGTCGCG |
| *pgaD* | *pgaD*-qRT-F | GCTTCAATTCGCACCTGATAC |
|  | *pgaD*-qRT-R | TTCAACAGTTCCCAGAGCG |
| *xagA* | *xagA*-qRT-F | CAACGGGTTTTGCAGATCAG |
|  | *xagA*-qRT-R | ATTCGGGTGATACGTCTTGC |
|  | *xagA*-*Sal*I-F | AAAGTCGACACCTTGCTGCAGCTATGCG |
|  | *xagA*-del-R | GTACAGCGTGGACAACTGCATGCAAGCGCCGATGCCAGAG |
|  | *xagA*-del-F | CTCTGGCATCGGCGCTTGCATGCAGTTGTCCACGCTGTAC |
|  | *xagA*-*Hin*dIII-R | TTAAGCTTCAACCCGAAGGTGGCCAGA |
| *xagB* | *xagB*-qRT-F | CACTTCGGGTTGCAATTCATC |
|  | *xagB*-qRT-R | CGTAACTATCCAGATCGCCG |
|  | *xagB*-*Eco*RI-F | AAGAATTCAATAACGATGCGCCGGTTGA |
|  | *xagB*-del-R | ACTGCGATACAGATGCACCGGTGATGACGCCTGCCGAAAC |
|  | *xagB*-del-F | GTTTCGGCAGGCGTCATCACCGGTGCATCTGTATCGCAGT |
|  | *xagB*-*Hin*dIII-R | TTAAGCTTCAACCTGCGCCTGCAAC |
| *xagC* | *xagC*-qRT-F | TTCCTGTTGCCGATCCTTG |
|  | *xagC*-qRT-R | TCCGCAGATAAGTGAACGC |
| *xagD* | *xagD*-qRT-F | GGTATTGGGCAGCGACAG |
|  | *xagD*-qRT-R | AGGTTGCTTGATCGGTGG |
| *gumB* | *gumB*-qRT-F | TTGATCTGCTCGATGACACTG |
|  | *gumB*-qRT-R | GGAACACCTTGACCAGCA |
| *gumC* | *gumC*-qRT-F | CTTTGGGACTTATCTTTGGCG |
|  | *gumC*-qRT-R | TTTCCTGCCCCTTGACG |
| *gumD* | *gumD*-qRT-F | TTGAACCATCTGCATACCCAG |
|  | *gumD* -qRT-R | ACCAGATTCATACCAACCCAC |
| *gumK* | *gumK* -qRT-F | AGTCGGTTCGATGCTGTTC |
|  | *gumK* -qRT-R | TCATTTCGCCATAGACCACG |
| 16S | 16S-qRT-F | CGCAAGACTGAAACTCAAAGG |
|  | 16S-qRT-R | AAGGCACCAATCCATCTCTG |
|  | 16S-RNA-F | CTCTACGCATTTCACTGCTACACC |
|  | 16S-RNA-R | CGCTAATACCGCATACGACCT |
| *gus* | GUS-1 | GCAACTGGACAAGGCACT |
|  | GUS-2 | GCGTCGCAGAACATTACA |

^a^ Restriction enzyme recognition sites are underlined
